# A novel SNP analysis method to detect copy number alterations with an unbiased reference signal directly from tumor samples

**DOI:** 10.1186/1755-8794-4-14

**Published:** 2011-01-26

**Authors:** Alex Lisovich, Uma R Chandran, Maureen A Lyons-Weiler, William A LaFramboise, Ashley R Brown, Regina I Jakacki, Ian F Pollack, Robert W Sobol

**Affiliations:** 1 Department of Biomedical Informatics, University of Pittsburgh School of Medicine, Pittsburgh, PA, 15213, USA; 2 Department Department of Pathology, University of Pittsburgh School of Medicine, Pittsburgh, PA, 15213, USA; 3 Department of Neurosurgery, University of Pittsburgh School of Medicine, Children's Hospital of Pittsburgh, Pittsburgh, PA 15213, USA; 4 Department of Pharmacology & Chemical Biology, University of Pittsburgh School of Medicine, Pittsburgh, PA, 15213, USA; 5 Clinical Genomics Facility, University of Pittsburgh School of Medicine, Pittsburgh, PA, 15213, USA; 6 University of Pittsburgh Cancer Institute, Hillman Cancer Center, Pittsburgh, PA, 15213, USA; 7 Division of Pediatric Hematology/Oncology, Children's Hospital of Pittsburgh, Pittsburgh, PA, 15213, USA; 8 Department of Pediatric Neurosurgery, Children's Hospital of Pittsburgh, Pittsburgh, PA, 15213, USA; 9 Department of Human Genetics, University of Pittsburgh Graduate School of Public Health, Pittsburgh, PA, 15213, USA

## Abstract

**Background:**

Genomic instability in cancer leads to abnormal genome copy number alterations (CNA) as a mechanism underlying tumorigenesis. Using microarrays and other technologies, tumor CNA are detected by comparing tumor sample CN to normal reference sample CN. While advances in microarray technology have improved detection of copy number alterations, the increase in the number of measured signals, noise from array probes, variations in signal-to-noise ratio across batches and disparity across laboratories leads to significant limitations for the accurate identification of CNA regions when comparing tumor and normal samples.

**Methods:**

To address these limitations, we designed a novel "Virtual Normal" algorithm (VN), which allowed for construction of an unbiased reference signal directly from test samples within an experiment using any publicly available normal reference set as a baseline thus eliminating the need for an in-lab normal reference set.

**Results:**

The algorithm was tested using an optimal, paired tumor/normal data set as well as previously uncharacterized pediatric malignant gliomas for which a normal reference set was not available. Using Affymetrix 250K Sty microarrays, we demonstrated improved signal-to-noise ratio and detected significant copy number alterations using the VN algorithm that were validated by independent PCR analysis of the target CNA regions.

**Conclusions:**

We developed and validated an algorithm to provide a virtual normal reference signal directly from tumor samples and minimize noise in the derivation of the raw CN signal. The algorithm reduces the variability of assays performed across different reagent and array batches, methods of sample preservation, multiple personnel, and among different laboratories. This approach may be valuable when matched normal samples are unavailable or the paired normal specimens have been subjected to variations in methods of preservation.

## Background

DNA copy number alterations (CNA) including sequence amplifications and deletions can cause oncogene activation or reduced tumor suppressor gene function associated with the emergence of cancer [1,2]. Recent advances in microarray technology including high density SNP (single nucleotide polymorphism) profiling have provided a novel approach to evaluate CNA across the genome of patient tissue specimens as a potential diagnostic tool for tumor classification. However, detection of a copy number amplification or deletion is critically dependent on: 1) interrogation of SNPs and/or nonpolymorphic markers at a frequency sufficient to identify changes in specific genes, and 2) accurate detection of gene copy number in tumor tissues [3]. Evolution of arrays with increasing densities of SNPs has mitigated the first factor but the ability to accurately detect CNA remains a formidable challenge. Specifically, the signal-to-noise ratio of copy number intensity from tumor samples is markedly decreased (or inferior) compared to that obtained from ideal samples such as homogeneous cell lines or blood samples. These differences are likely associated with cellular heterogeneity e.g. normal cells mixed with tumor cells, infiltrating inflammatory cells and/or the presence of proliferating, apoptotic or necrotic cells. The variability is compounded further by sample preservation e.g. formalin-fixed paraffin embedded (FFPE) exhibit higher variability than frozen samples. Thus, there are substantial methodological hurdles to be resolved before CNA values from SNP arrays can be integrated into diagnostic evaluation of tumor samples.

An ideal experimental comparison for tumor copy-number evaluation comprises paired, normal tissue samples processed simultaneously with the tumor specimens thereby eliminating technical variability from personnel and batch effects. One additional advantage to paired comparisons is the suppression of CN aberrations present in both normal and tumor tissues. In practice however, paired tissue samples are rarely available and acquisition of paired normal tissue does not typically fall within the purview of therapeutic surgical intervention. Consequently, normal CNA profiles are often derived from a variety of normal specimens accumulated in a laboratory and often include archived FFPE specimens. Otherwise, investigators must rely on data from other laboratories employing the same CNA platform and/or by comparison to a publicly accessible database. The variability inherent in these other data sources further complicates interpretation of tumor sample CNA results. To address this issue, we have developed a SNP CN detection algorithm that derives a reliable copy number reference signal specifically from tumor samples thereby eliminating the need for a normal reference set. Validation tests indicated that this approach achieves a signal-to-noise ratio surpassing that obtained by other techniques including paired normal reference sets.

## Implementation

### General Approach

Reducing the noise level by exploiting similarities between the test and reference set has been successful in at least two empirical studies [4,5]. In one study [4], the entire data set (test as well as normal data if available) is acquired in the lab to build a reference set. While noise reduction is achieved, this approach suppresses the CNA common for all samples in a data set, making it impractical for systematic CN aberration analysis. The second employs a CN detection package in CNAG [5], selecting the best from the pool of normal samples based on similarity of correlation function between a particular test sample and a given set of normal samples. While the noise level is somewhat reduced, testing this approach in CNAG as well as in our own implementation of a similar selection algorithm (data not shown) shows that sometimes, the reference set selected using this method may be very small (up to 1 - 2 samples) and therefore any CNA present in a normal reference will introduce false CNA in the tumor CN signal. Nevertheless, exploiting the natural similarity between test and reference set is an attractive approach if implemented carefully.

### Methodological Considerations

Consider a scenario where the reference signal is computed as an average (median) of tumor intensity signals instead of traditional average of normal reference samples intensities. Such signal would preserve the high frequency component common for all tumor samples in a given batch and therefore if used as a denominator in computing the (log2) ratio in place of the standard reference would decrease the noise this high frequency component is responsible for. However this reference signal would carry the CN variations common for the given tumor sample set and therefore, if used unmodified, would suppress these common variations for each particular tumor sample where they are present and cause the false CN variations for the rest of the tumors. To overcome this problem, we can compute the (log2) ratio between this synthetic reference signal and normal reference set based signal (treating these two references as a test/normal pair) and then employ the mean preserving segmentation or filtering technique to detect the common CN variations. If, then, we use the information about the position and amplitude of the common CN variations to modify the tumor based reference signal in such a way that these common CN variations go away when tested against the traditional reference signal, we would obtain a synthetic reference signal which has two key properties:

a) Its high frequency component is highly correlated with the one common for all tumor samples in the given batch and therefore the noise in (log2) ratio between given tumor raw CN and the reference signal is reduced.

b) The reference signal does not contain the CN aberrations common for the part or the whole tumor sample set, which means that when each individual tumor sample is compared to the reference signal, the aberration will be picked up for this particular tumor.

**Figure 1:**
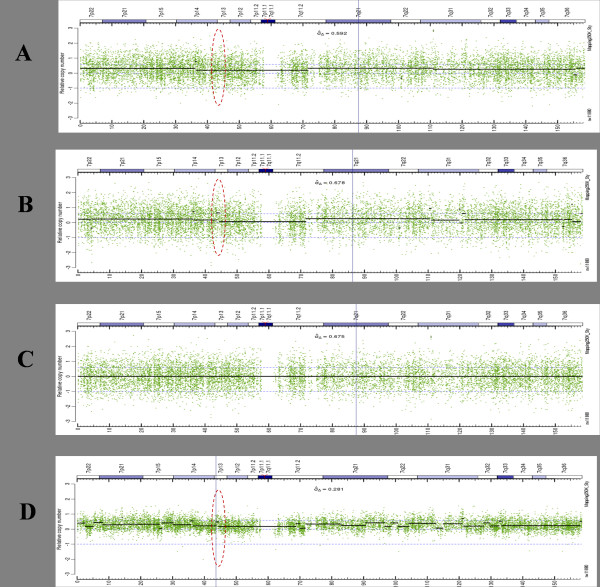
**Virtual reference signal and noise reduction**. A: The raw CN and segmentation results for sample 08_0015 using HapMap as a reference set. Note the absence of the copy number amplification at 43Mb (red dotted oval). B: The raw CN and segmentation results of the average signal computed over the whole tumor data set vs. average signal computed over the HapMap data set. Note the multiple CN events detected (e.g., at 43Mb) due to common CN aberrations in tumor samples. If used as a reference signal, this aberration would cancel out the real CNV in corresponding tumor samples. C: The VN-corrected tumor data set based average signal. Aberrations are removed using the VN algorithm, and the resulting signal can be used as an unbiased reference for further processing. D: The raw CN and segmentation results using the VN based reference signal from C. Note the detection of the copy number amplification (red dotted oval) at 43Mb that would be lost using a HapMap based reference signal.

The whole process is illustrated on the Figure. 1, where Figure 1A shows the raw CN signal for a single FFPE tumor sample obtained using the traditional reference signal based on frozen HapMap normal set, the Figure 1B shows the raw CN segmentation results for log2 ratio between the tumor and normal based reference signals and Figure 1C shows the log2 ratio between the final transformed synthetic reference and normal based reference signals. Note how the common tumor CN variations got removed from the synthetic reference set. The Figure 1D shows the raw CN and the segmentation results for this tumor sample using the synthetic reference and illustrate significant (> 2 times) noise reduction compared with traditional approach (Figure 1A). The original normal reference set is used here as a template to construct an optimal reference signal and will be referred to further as a template reference set when applicable.

### Unbiased Reference Signal Restoration

Depending on the signal-to-noise ratio between averages, we can employ two basic strategies for detecting and correcting for shifted segments. If the signal-to-noise ratio is high enough so that the false positive (FP)/false negative (FN) detection is low, we can use the direct segment detection, thus achieving the highest possible resolution. Shifting the segments back is a problem requiring detection of points of discontinuity of signal and calculating the mean over the segment. There are very few efficient segmentation algorithms available [6-8], which preserve a segment mean and therefore can be used to accomplish the task. The performance of a segmentation algorithm in this case should be higher than on a single sample, as the ratio of averages would not include the instrument noise.

If the noise level is too high so that FP/FN segment detection is likely, then instead of direct segment detection, the low-pass filtering or window smoothing is used for detecting the shift pattern, followed by correcting the original average intensity signal for an abovementioned shift pattern. With a proper choice of a smoothing window width, the problem of FP/FN detection presented in the first method is eliminated. In practice, the width of a smoothing window is comparable with one used for smoothing in standard methods (e.g., 10 - 30 data points). For comparatively rare CN events (i.e. less than 25 -30% of test samples), the smoothing described above does not substantially alter the final spatial resolution, as the CN aberrations will be suppressed by the same 25 - 30% thus preserving the high frequency component.

### Data Set

Tumor DNA was obtained from a de-identified series of 25 pediatric malignant glioma tissue samples (Children's Oncology Group ACNS0423 study). Tissue accrual for the current study was coordinated by the Pediatric Branch of the Cooperative Human Tissue Network (CHTN). This study was approved by Institutional Review Board (IRB) for the University of Pittsburgh. Patients included children with malignant gliomas arising outside the brainstem treated with surgery, field irradiation, and chemotherapy using temozolomide administered on a daily schedule during irradiation, and in conjunction with lomustine after irradiation. Eligibility for the study required institutional IRB approval as well as central review of the histopathology, and confirmation of a diagnosis of glioblastoma, anaplastic astrocytoma, or gliosarcoma.

Samples were processed and data was collected using the Affymetrix Mapping 250K Sty microarray chip using standard procedures [9] that have been optimized in the UPCI Clinical Genomics Facility (detailed methods are described in Additional file 1). All data have been deposited in the Gene Expression Omnibus (Accession number GSE25589). Due to the specifics of the sample collection protocol, paired normal samples (blood DNA) were not available for analysis. Also, because of the sample preservation method (FFPE), using the HapMap data as a normal reference set proved difficult due to the very high noise level, likely from the difference in preservation protocols between the glioma samples (FFPE) and HapMap (frozen). We therefore used an in-house processed FFPE reference set, which was used to validate the results obtained through the VN approach, as described. We believe that having a sample set without an ideal reference set is a universal problem in cancer translational research and highlights the potential usefulness and practicality of the VN method we describe.

### Generating the VN Reference Signal

In preparation for generating the VN reference signal, the intensities of test and reference samples were preprocessed within the aroma.affymetrix framework [10] using the latest CRMAv.2 method [11,12]. During the preprocessing step, the sample intensities were corrected for allelic crosstalk, normalized for nucleotide-position probe sequence effects, summarized and finally corrected for the PCR fragment length. We implemented our own GC content correction using the approach described by Diskin and colleagues [13], modifying it slightly so the regions of high amplification/deletion were also included into the GC wave parameter estimation. All processing was done on the total intensities and aimed to total raw CN estimates, but the results are also applicable for allele-specific analysis.

### Algorithm Description

Let *I^t^_i_(p) *and *I^r^_j _(p) *be the signal intensities for test (*t*) and reference set (*r*), where *i *= 1.N and *j *= 1. M is a sample number for test (*i*) and reference (*j*) sets respectively and *p *is a genomic position. For the segmentation-based version of the algorithm, the VN reference signal is generated as follows:

Compute the average signal for tumor test set and the normal reference set separately, so

(1a) $$I^{t}avg\;\left(p\right)=median\left(I^{t}{}_{i}\left(p\right)\right)$$

and

(1b) $$I^{r}avg\;\left(p\right)=median\left(I^{r}{}_{i}\left(p\right)\right)$$

where *I^t^avg *and *I^r^avg *are intensity medians computed for each chromosomal position *p *over the whole test and reference sets respectively

Compute the (log2) ratio of two averages producing the raw CN average signal

(2) $$CNavg=log2\;(I^{t}avg/I^{r}avg)$$

Perform the GC correction on CNavg signal using the modified PennCNV [13].

Perform the segmentation of the average signal using one of the segmentation algorithms which preserve the mean level of each signal [6-8], so

(3) $$S\;\left(p\right)=M\left(CNavg_{s}\left(p\right)\right)$$

where *S(p) *is a piece-wise constant function of the chromosomal position and *M(CNavg_s_(p)) *is a mean of (log2) ratio of two averages from (2) at the segment *s *for the range of chromosomal positions *p*.

Using the segmentation results, modify the average tumor set intensity signal in such a way that the regions of the segmentation results deviating from the baseline (zero for log2 ratio) would get shifted back to the baseline, producing the virtual reference signal:

(4) $$I^{vn}\left(p\right)=\;I^{t}avg\left(p\right)*\;2^{-S(p)}$$

where *p *is a chromosomal position, *I^vn^(p) *is a virtual reference intensity signal, *I^t^avg *is an average intensity of tumor samples and *S(p) *is a segmented average CN from (2)

Use the resulting modified tumor average intensity signal as a new (virtual) reference when generating the raw CN signal for each tumor sample, i.e.

(5) $$rawCN_{i}=log2\left(I_{i}/I_{vn}\right)$$

where the rawCN_i _is a raw CN signal for sample I, I_i _is an intensity for tumor I and I_vn _is an intensity of the modified tumor intensity average signal (the virtual reference) from equation (4).

As was mentioned before, one of the major drawbacks of using the segmentation technique in obtaining the reference signal is that the erroneous (false positive) detection of a segment would lead to a corresponding false peak in all samples run against such a reference set. A more conservative approach for eliminating such a possibility involves filtering the log2 ratio and using this filtered signal in place of a segmented signal. In this case the expression for S(p) (3) is given by the formula:

(3a) S(p)=F(CNavg,w)

where *F *is a filtering operator (moving Gaussian smoothing window for example) and *w *is a width of smoothing window.

The trade-off of this approach is that the loss of resolution due to the filtering leaves the narrow peaks in the virtual reference signal uncorrected, which in turn suppresses the peaks in each corresponding test sample exhibiting such a peak. In practice we found that generating the virtual reference based on a set of normals from the same lab requires the same filtering window width as when using a standard approach. Moreover, the peak suppressing effect largely goes away when the CN aberration event is not present in all test samples, which is a typical situation. In practice, the peak suppression of very small CN regions did not exceed 15% - 20% and in most instances was undetectable, which allows using the noise reduction obtained through the VN to full advantage.

### Methods for Implementation and Validation

The algorithm was implemented in R as an add-on to the aroma.affymetrix extendable open source CNV analysis package [10]. The main workflow remained comparable to the conventional scenario of using an unpaired reference set, with the major exception relating to the method of obtaining the reference signal. Instead of using the average of a normal reference set signal, the VN reference is obtained as described above. Another difference with the aroma.affymetrix workflow is a correction for GC content which is implemented based on ideas from the PennCNV package [14] with a modification allowing the inclusion of the amplified/deleted regions into the GC curve fitting process, thus improving the robustness of correction in case the samples have a high percentage of CN aberrations. The VN reference generation algorithm also uses this version of the GC correction. While the authors of the aroma.affymetrix package suggest that for high-density chips (250K and above) GC correction is not necessary, we found it to be very beneficial in our case, especially during processing of FFPE samples using the frozen reference set as a template for VN.

**Figure 2:**
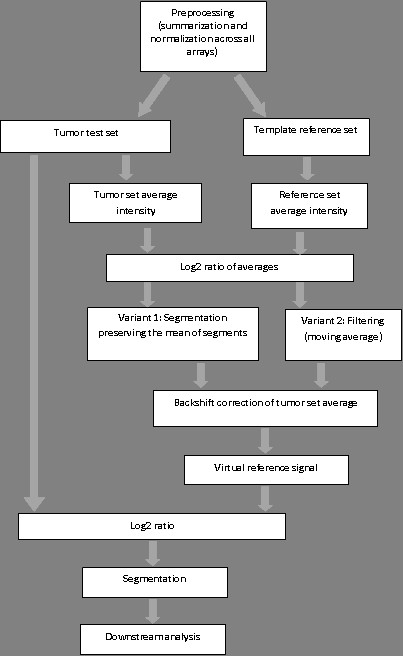
**Algorithm block scheme**.

The segmentation of the resulting raw CN signal as well as a segmentation-based version of the VN algorithm was implemented using the Sparse Bayesian Learning-based GADA package [7]. The block-scheme of the algorithm is presented in Figure 2.

We assessed the validity of this method in several ways. First, using a data set from nine tumor/normal pairs (available from Affymetrix; see Additional file 1), we compared the validity and performance of the method with the standard unpaired and paired approach as outlined below. This data set was chosen for a preliminary test due to the high signal-to-noise ratio as well as known SNP CN aberration positions. Next, the level of noise and the quality of segmentation of the glioma samples under consideration were assessed using the HapMap normal samples as a reference set. A conventional CN analysis using the tumor and in-house lab FFPE sample set (5 normal FFPE blood samples) was then used to validate the VN algorithm on the HapMap results. We also constructed a second VN reference signal using the same normal in-lab sample set as a template and the results were compared to assess the usability of a VN with Hapmap. Further, the filtering version of the VN algorithm was applied to generate a reference signal based on the HapMap template and the gain in signal-to-noise ratio was gauged.

To validate the VN results directly, genomic DNA was analyzed using Applied Biosystems TaqMan^® ^Copy Number Assays (chromosome 4: Hs04800686_cn; chromosome 15: Hs05319670_cn, Hs01691525_cn) with the Applied Biosystems StepOnePlus system. Each sample was analyzed in quadruplicate as per the manufacturer's instructions (ΔΔC_T _method). The data was imported into Applied Biosystems CopyCaller™ software and the copy number for chromosomes 4 and 15 was calculated. The results are shown as an average of three independent experiments +/- S.E.M.

## Results

### Validation using Tumor/Normal Paired Samples

**Figure 3:**
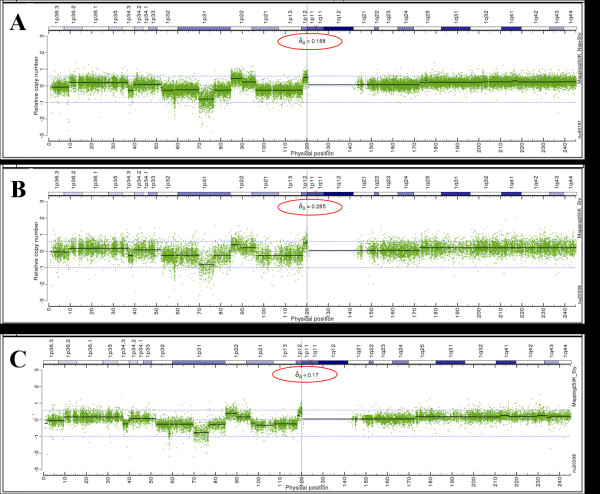
**Validation of VN-based processing for sample CRL-2324, Chr.1**. A: Traditional paired processing, nine tumor-normal pairs. B: Traditional unpaired processing using the HapMap reference set. The SD is roughly 1.6 times higher than for paired processing, C: VN algorithm based processing (HapMap data set as a template). Note the SD is even less than for paired processing (by ~10%) which can be explained by elimination of the instrument noise in the virtual reference signal, which is essentially a modified average of all tumor samples. The SD is reduced ~ 1.7 times compared to the processing using the HapMap reference set.

Nine paired tumor and normal samples with high intra-specimen tissue homogeneity and copy number signal intensity (Affymetrix: see see Additional file 1) were used as "ideal" sample set to validate the Virtual Normal algorithm developed in this study. These paired samples were combined with the normal HapMap samples in multiple combinations to emulate scenarios of data acquisition and processing. Specifically, processing strategies were compared using (1) paired analysis of the nine optimal tumor-normal pairs; (2) non-paired analysis of the nine tumors using HapMap normals as the reference set; and (3) the reference signal generated by the Virtual Normal algorithm from the nine tumor samples with the HapMap normals as a baseline. The results from processing sample CRL-2324, Chr.1 are presented in Figure 3 (A-C: sigma values above each graph represent the standard deviation (SD) of log2 ratio). The Virtual Normal algorithm provided the lowest noise level among all three approaches while yielding comparable results for raw CN and segmentation.

### Application to Pediatric Glioma Using FFPE Samples

**Figure 4:**
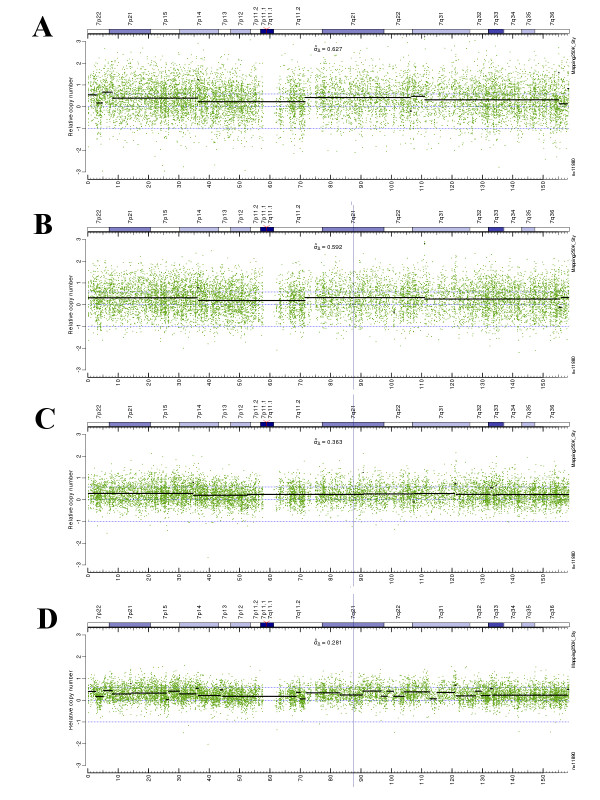
**Comparison of various approaches to generate the reference signal and its contribution to the noise level using sample 08_0015, Chr.7 as an example**. A: In-lab frozen blood normals based reference (15 samples). B: HapMap normals based reference (49 samples). C: In-lab FFPE normals reference (5 samples). D: The VN generated reference signal, HapMap template based.

**Figure 5:**
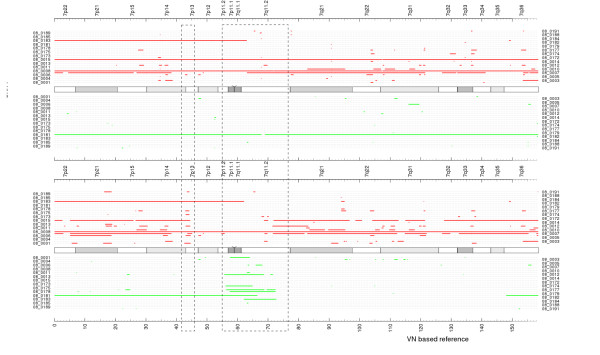
**Comparison of detection results on Chr. 7. The red and green segments represent detected amplifications and deletions, correspondingly**. A: Using FFPE in-lab normals. B: Using the VN reference set. The 7p13 region was detected using VN algorithm only.

We compared the results of processing with the Virtual Normal algorithm to traditional approaches utilizing different reference sets including: (1) a set of normal frozen tissue samples processed in the laboratory; (2) the HapMap reference set obtained from frozen tissue; (3) samples of normal tissue after formalin fixation and paraffin embedding (FFPE) and (4) the VN reference set with HapMap data as a baseline. A comparison of the raw CN estimates along with segmentation results for sample 08_0015 on Chr.7 is presented in Figure 4. These data demonstrate that inter-laboratory variability (HapMap), sample preservation (FFPE vs. frozen) and inter-batch variability (in-lab FFPE reference set) all contribute to increased noise that is further compounded when admixtures of these sample types are combined. It is important to note that the virtual reference signal constructed from the tumor data set using the VN algorithm achieved the lowest noise level of the four methods (panel D). Concordance between segmentation results for the FFPE reference set and the VN reference set was assessed by generating plots of segmentation results corresponding to +/- 0.2 threshold in log2 ratio. Summary plots of segments detected on Chr.7 compared VN with the best results achieved by traditional methods (Figure 5) demonstrating high concordance between the VN approach and the best traditional methods.

**Figure 6:**
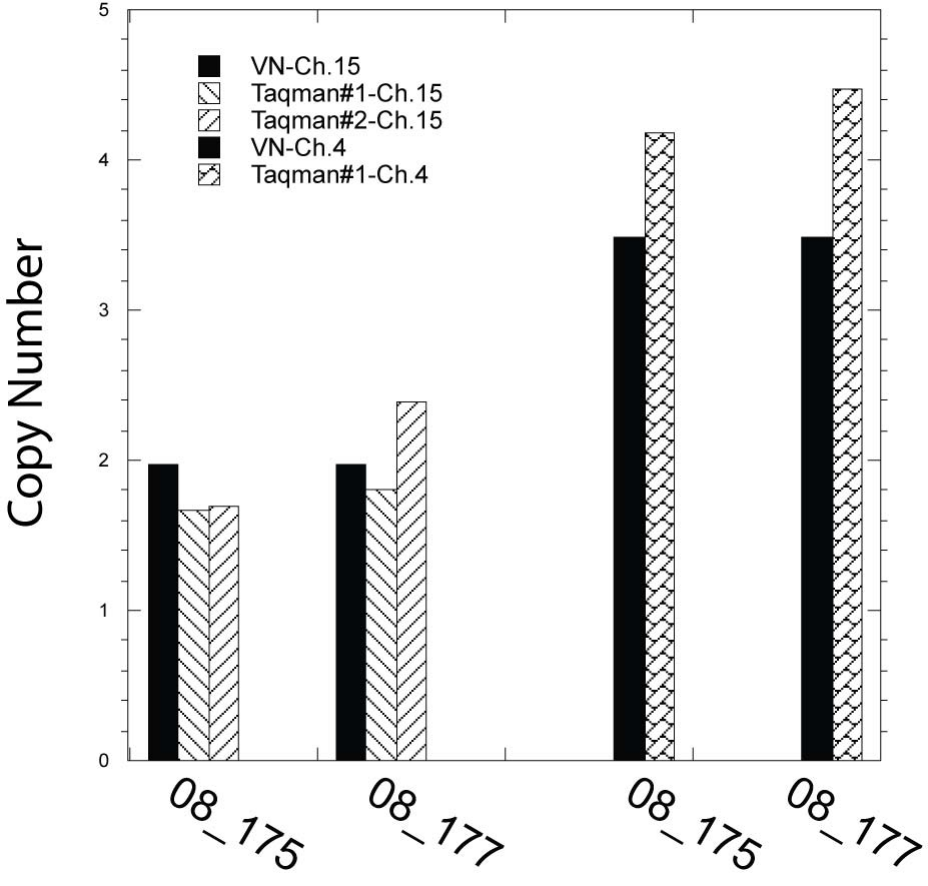
**Copy number validation**. Copy number validation as determined by Taqman Copy number analysis for samples 08_175 and 08_177 at Chr.4 and Chr.15, as indicated.

Segmentation plots of all chromosomes demonstrated high concordance between the VN analysis and the best traditional approaches (see Additional file 1). Comparison of raw copy number standard deviation and corresponding signal-to-noise ratio averaged over all glioma samples/chromosomes for the 4 processing scenarios are summarized in Table 1. Use of the Virtual Normal method increased the signal-to-noise ratio from unacceptable (< 1) when processing FFPE tissue samples using the frozen reference set to a reliable level (~2) with minimal pre-segmentation signal smoothing of the VN reference signal. Validation of the analysis is demonstrated in Figure 6 including measurement of copy number in a region of chromosome 4 and 15. The region of Chr.4 was determined to be elevated in several glioma tumor samples whereas all samples were determined to have a copy number of 1.97 for this region of Chr.15 using the VN approach. Copy number changes determined using our VN analysis were confirmed using a PCR-based copy number analysis approach. The VN assay indicated that a specific 2.9 Mb region of Chr.4 was amplified with a raw CN average of 3.48 for samples 08_0175 and 08_0177. PCR analysis of this region computed a copy number of 4.18+/- 0.45 and 4.47+/-0.58 reinforcing the sensitivity of copy number determinations obtained using the VN approach.

**Table 1:** Comparison of a standard deviation (SD) and signal to noise (S/N) ratio for a raw CN signal obtained using different types of references. Here SD is an average of all SDs obtained for each raw CN segmented region in log2 scale, and S/N is computed as a ratio of the difference between the raw CN values corresponding to CN = 3 and CN = 2 in log2 scale and the SD value defined above

FFPE Tumor vs:	Blood normals (frozen)	HapMap (frozen)	FFPE normals	VN based
SD (log2)	0.627	0.592	0.363	0.281

S/N ratio for CN = 3 (log2)	0.797	0.845	1.377	1.78

### Application to higher density arrays

Initially we were motivated to develop this algorithm for the particular study on the Affymetrix 500K platform due to the absence of the adequate normal FFPE reference set, i.e. under the most unfavorable conditions. However, the algorithm is generic enough to be applied to a wide range of platforms which utilize the normal sample set to generate a reference signal. In particular, it was successfully tested during the renal carcinoma studies (frozen tumor and normal samples) performed on the Affymetrix Genome-Wide 6.0 platform (see Additional file 1, Figure S7 A, B and C). Here we show the exemplary raw CN and segmentation processing results for a renal carcinoma sample on Chr. 2 which utilize three different sources of the reference signal: a) HapMap normal set (Additional file 1, Figure S7a); b) in-lab normal set (Additional file 1, Figure S7b) and c) VN using HapMap as a template (Additional file 1, Figure S7c). The reference signal generated using VN algorithm allowed to achieve the best signal-to-noise ratio compared to the traditional methods even when in-lab normal reference set with matching preservation protocol was available.

## Conclusions

We have demonstrated that it is possible to construct a reference signal directly from the tumor sample intensities using the normal sample reference set as a template and that such a reference has the properties to minimize the noise in the resulting raw CN signal. The proposed algorithm allows minimizing the negative effect of inter-batch or inter-lab variability and is generic enough to be applicable to a variety of platforms which utilize the reference signal for processing. When the test sample preservation protocol differs from the one for the available normal reference set or when the in-lab reference set is unavailable, the described approach could likely be an ideal option to produce reliable total CN estimates. We did not investigate the applicability of the method for allele-specific CN estimates, but it's likely it could be used in such instances as well.

We have discussed the tradeoffs between segmentation based and filter based VN versions. Our primary goal was to accommodate the different signal-to-noise ratios between two average intensities so to achieve the maximum spatial resolution while minimizing FP/FN events. We believe, that using the wavelet based filtering similar to CDF 5/3 [15,16], would help to achieve an optimal compromise between a low FN/FP rate as for filtering based VN and the highest possible spatial resolution. We are working on incorporating such filtering as an option for the VN algorithm.

The recent trend in CNV detection algorithms is to use the total and allele-specific CN estimates and the genotyping call results together to obtain the more reliable CN event detection [17]. We plan to work on such extension of the aroma.affymetrix package including the VN into the processing pipeline in a near future.

Finally, during evaluation, the VN algorithm demonstrated a better signal-to-noise ratio than paired analysis. Still, in most cases, the paired analysis is preferable as it allows exclusion of CN aberrations that are not disease-specific. However, in rare cases, the paired normal may have disease specific aberrations if obtained in close proximity to the tumor. We suggest that using both methods applied to the same dataset would provide more information on the disease related CN aberrations, than each method applied alone.

The algorithm was implemented in R language as an add-on to the open source aroma.affymetrix package but can be easily adapted for use with other packages. The algorithm source code as well as the GC correction module along with necessary modifications in aroma.affymetrix package is readily available for download at the following URL - http://lnx02.dbmi.pitt.edu/download/Aroma%20Extensions.zip.

## Availablility and Requirements

Project name: Virtual Normals

Project home page: http://lnx02.dbmi.pitt.edu/download/Aroma%20Extensions.zip

Operating system(s): Platform independent.

Programming language: R

Other requirements: R 2.11 or higher, aroma.affymetrix 1.5.0 or higher.

License: Please contact corresponding author (RWS) for commercial license. No license required for academic use.

Any restrictions to use by non-academics: License needed for commercial use.

## Abbreviations

VN: virtual normals; (algorithm described herein) CN: copy number; CNA: copy number alterations; SNP: single-nucleotide polymorphism; FFPE: formalin-fixed paraffin embedded sample preservation protocol; FP: false positive; FN: false negative; SD: standard deviation; GC, GC content: guanine-cytosine content; GC wave: variation in hybridization intensity associated with the GC content

## Competing interests

The authors declare that they have no competing interests.

## Authors' contributions

AL designed the VN algorithm. AL, URC, and RWS wrote the paper. AL implemented the algorithm. ARB conducted Taqman validation experiments. MAL-W and WALF purified the genomic DNA, processed all microarrays used in the experiments and performed primary analysis of the data. RIJ and IFP provided the tumor tissue. All authors read and approved the manuscript.

## Pre-publication history

The pre-publication history for this paper can be accessed here:

http://www.biomedcentral.com/1755-8794/4/14/prepub

## Supplementary Material

Additional File 1 **Definitions, additional methods, a description of the paired tumor/normal data set, Heat Maps, Segmentation plots and a comparison of raw CN and segmentation results**. The file contains: a) A set of definitions for some terms used throughout the text. b) A detailed method for the Affymetrix 250K Sty assay for formalin fixed, paraffin embedded tissue (FFPET) samples. c) Information on the paired tumor/normal data set used. d) References Cited in the Additional file. e) Heat Maps for the (1) in-lab FFPE normals reference, (2) the VN reference based on in-lab FFPE normals data set and (3) VN reference based on HapMap data set. f) Segmentation plots for the (1) in-lab FFPE normals reference, (2) the VN reference based on in-lab FFPE normals data set and (3) VN reference based on HapMap data set. g) A figure showing a comparison of raw CN and segmentation results (Affymetrix 6.0 array) for a renal carcinoma sample on Chr.2 utilizing three different sources of the reference signal. Click here for file
