# Supplementary material for: A novel SNP analysis method to detect copy number alterations with an unbiased reference signal directly from tumor samples
Source: BMC Med Genomics. 2011 Jan 26;4:14. doi: 10.1186/1755-8794-4-14 (PMC3041647; doi:10.1186/1755-8794-4-14)
Supplement: Additional File 1 — Definitions, additional methods, a description of the paired tumor/normal data set, Heat Maps, Segmentation plots and a comparison of raw CN and segmentation results. The file contains: a) A set of definitions for some terms used throughout the text. b) A detailed method for the Affymetrix 250K Sty assay for formalin fixed, paraffin embedded tissue (FFPET) samples. c) Information on the paired tumor/normal data set used. d) References Cited in the Additional file. e) Heat Maps for the (1) in-lab FFPE normals reference, (2) the VN reference based on in-lab FFPE normals data set and (3) VN reference based on HapMap data set. f) Segmentation plots for the (1) in-lab FFPE normals reference, (2) the VN reference based on in-lab FFPE normals data set and (3) VN reference based on HapMap data set. g) A figure showing a comparison of raw CN and segmentation results (Affymetrix 6.0 array) for a renal carcinoma sample on Chr.2 utilizing three different sources of the reference signal. [file 1755-8794-4-14-S1.PDF]

Supplementary Material for:

A novel SNP analysis method to detect copy number alterations with an unbiased reference signal directly from tumor samples

Definitions

Log2 ratio: if I1 is an intensity signal from array 1 and I2 is an intensity signal from array 2 then log2=log2(I1/I2).

Standard deviation (SD): the square root of a variance; the variance is a sum of squared differences between the signal value at a given point and a signal mean.

Unpaired data set: the tumor (test) and normal samples were collected from different subjects. In this case the raw copy number (CN) for each tumor (test) sample is computed using the (log2) ratio between a given tumor (test) sample intensity and the average intensity of all normal samples.

Paired data set: the tumor (test) and normal samples were acquired from the same subject. In this case the raw CN for each tumor (test) sample is computed using the (log2) ratio between given tumor (test) sample intensity and the normal sample intensity from the same subject.

Segmentation: assigning a constant value to the subset of adjacent signal data points based on some data points with similar criteria. The assigned value could be a mean or median (CBS, GADA) or the copy number (HMM).

Affymetrix 250K Sty Assay for Formalin Fixed, Paraffin Embedded Tissue (FFPET) Samples

In most cases, genomic DNA samples extracted from formalin fixed, paraffin embedded tissues (FFPET) are more fragmented than genomic DNA extracted from fresh frozen tissue counterparts (Lyons-Weiler, et al., 2008). Therefore, we use the Sty assay to process FFPET samples, as the 250K Sty assay preferentially cuts a greater amount of shorter fragment length SNP's when compared to the 250K Nsp assay. The number of PCR reactions was increased since FFPET samples consistently under-perform when compared to frozen tissue with regard to PCR yield (not shown). We routinely run 4 replicates of each FFPET sample.

DNA was extracted from FFPET samples as previously described (Lyons-Weiler, et al., 2008). One microgram of whole genomic DNA was digested according to a modified restriction digest protocol (Lyons-Weiler, et al., 2008) with Sty I and restriction digest buffer 3 (New England Biolabs, MA) to begin the Affymentrix 250K assay. In short, an excess of both the Sty I enzyme and buffer are used to digest the excess genomic DNA. Upon DNA digestion, the sample is concentrated to 20 µl. The digested DNA is then ligated according to the manufacturer's instructions (Affymetrix 500K assay protocol). The digested, ligated DNA is then diluted and each sample undergoes PCR using 4 PCR reactions instead of 3 per sample (Lyons-Weiler, et al., 2008). PCR purification was carried out using Agencourt AmpPure beads (Beckman Coulter) (Affymetrix® Cytogenetics Copy Number Assay User Guide). Seventy to ninety micrograms of labeled, fragmented DNA was then loaded onto the Affymetrix Gene Chip 250K Sty arrays. Samples were hybridized according to the manufacturers instructions for 16 hours at 48˚C with 60rpm rotation in the Gene Chip hybridization oven (Affymetrix). Quality Control metrics were generated for each array using the Affymetrix -AGCC software.

Paired tumor/normal samples

The paired tumor/normal data set used here was obtained from Affymetrix:
http://www.affymetrix.com/support/technical/sample_data/copy_number_data.affx

These are:
(CRL-2324D, CRL-2325D);
(CRL-5868D, CRL-5957D);
(CCL-256D, CCL-256.1D);
(CRL-2320D, CRL-2319D);
(CRL-2321D, CRL-2362D);
(CRL-2336D, CRL-2337D);
(CRL-2338D, CRL-2339D);
(CRL-2340D, CRL-2341D);
(CRL-2314D, CRL-2346D).

References Cited in the Supplement

Lyons-Weiler, M., Hagenkord, J., Sciulli, C., Dhir, R. and Monzon, F. (2008) Optimization of the Affymetrix GeneChip Mapping 10K 2.0 Assay for routine clinical use on formalin-fixed paraffin-embedded tissue, *Diagnostic Molecular Pathology*, **17**, 3-13.

Supplemental Figure Legends

Figure S1: Heat Maps - in-lab FFPE normals reference

The heatmap plots for each chromosome represent the segmentation results for the tumor set using the in-lab FFPE normals as a reference set. The red regions denote amplifications and the green regions denote deletions. The maximum red/green brightness corresponds to amplification = 2.5 (red) and deletion = 1.5 (green). The low threshold value was selected to take into account the contamination of samples by normal tissue, which caused the reduction of signal values corresponding to the copy numbers 1 and 3.

Figure S2: Heat Maps - VN reference based on in-lab FFPE normals data set

The heatmap plots for each chromosome represent the segmentation results for the tumor set using the VN algorithm and the FFPE normals data set as a template. The red regions denote amplifications and the green regions denote deletions. The maximum red/green brightness corresponds to amplification = 2.5 (red) and deletion = 1.5 (green). The low threshold value was selected to take into account the contamination of samples by normal tissue, which caused the reduction of signal values corresponding to the copy numbers 1 and 3.

Figure S3: Heat Maps - VN reference based on HapMap data set

The heatmap plots for each chromosome represent the segmentation results for the tumor set using the VN algorithm and the HapMap data set as a template. The red regions denote amplifications and the green regions denote deletions. The maximum red/green brightness corresponds to amplification = 2.5 (red) and deletion = 1.5 (green). The low threshold value was selected to take into account the contamination of samples by normal tissue, which caused the reduction of signal values corresponding to the copy numbers 1 and 3.

Figure S4: Segmentation plots - in-lab FFPE normals based reference

The segmentation plots represent the segmentation results displaying only the regions that fall outside the predefined threshold range. The figures represent results for the tumor set using the in-lab FFPE normals as a reference set. The red lines denote the amplifications and the green lines denote the deletion. The threshold for amplification/deletion corresponds to a copy number of 2.4 / 1.6. The low threshold value was selected to take into account the contamination of samples by normal tissue, which caused the reduction of signal values corresponding to the copy numbers 1 and 3.

Figure S5: Segmentation plots - VN reference based on in-lab FFPE normals data set

The segmentation plots represent the segmentation results displaying only the regions that fall outside the predefined threshold range. The figures represent results for tumor set processing using the VN algorithm and the FFPE normals data set as a template. The red lines denote the amplifications and the green lines denote the deletion. The threshold for amplification/deletion corresponds to a copy number of 2.4 / 1.6. The low threshold value was selected to take into account the contamination of samples by normal tissue, which caused the reduction of signal values corresponding to the copy numbers 1 and 3.

Figure S6: Segmentation plots - VN reference based on HapMap data set

The segmentation plots represent the segmentation results displaying only the regions that fall outside the predefined threshold range. The figures represent results for tumor set processing using the VN algorithm and the HapMap data set as a template. The red lines denote the amplifications and the green lines denote the deletion. The threshold for amplification/deletion corresponds to a copy number of 2.4 / 1.6. The low threshold value was selected to take into account the contamination of samples by normal tissue, which caused the reduction of signal values corresponding to the copy numbers 1 and 3.

Figure S7: Comparison of raw CN and segmentation results (Affymetrix 6.0 array) for a renal carcinoma sample on Chr.2 utilizing three different sources of the reference signal.
A: HapMap based normal reference set.
B: In-lab normal reference.
C: VN reference signal using HapMap as a template.

Figure S1

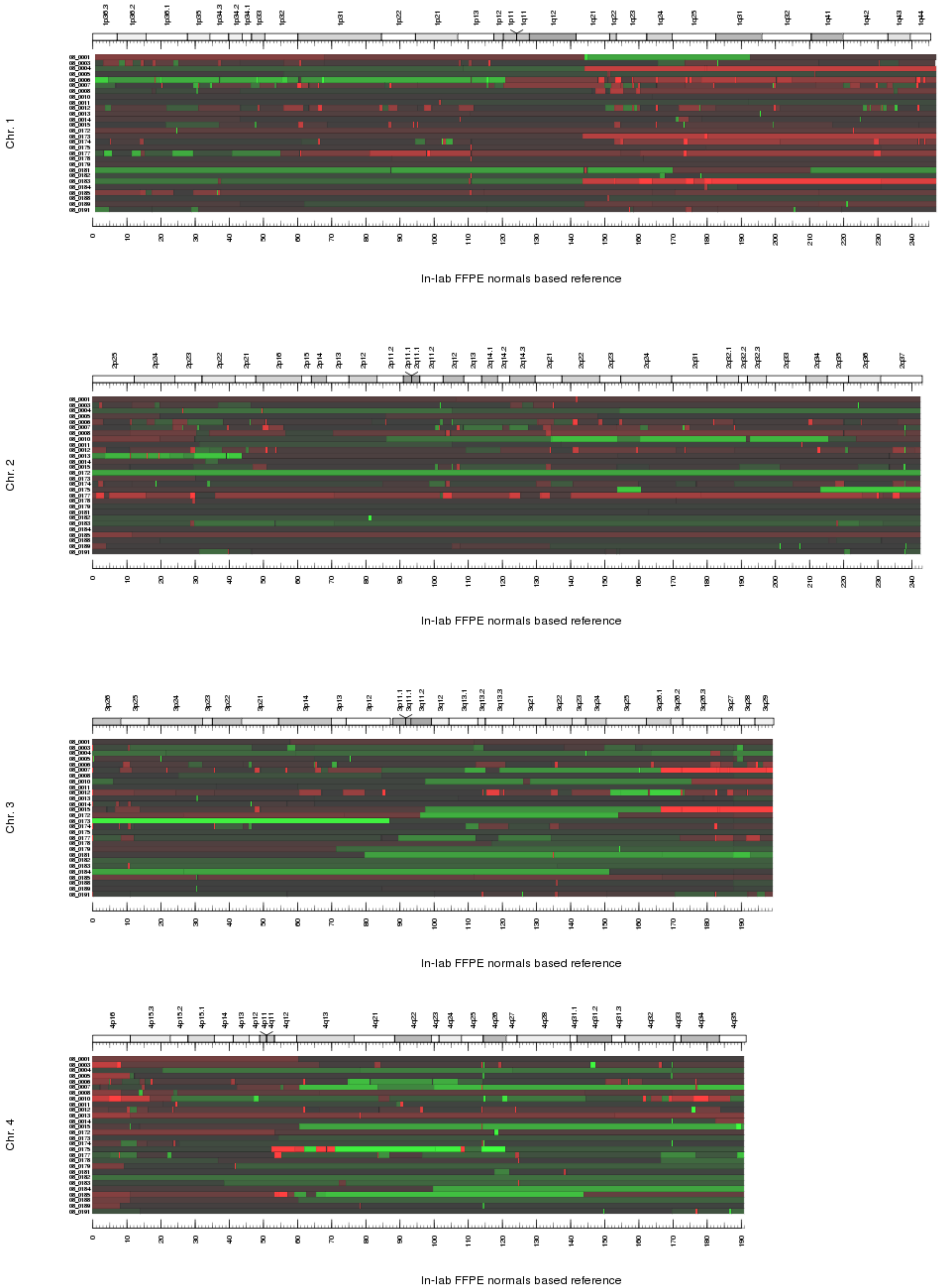

Chr. 1
In-lab FFPE normals based reference
Chr. 2
In-lab FFPE normals based reference
Chr. 3
In-lab FFPE normals based reference
Chr. 4
In-lab FFPE normals based reference

Chr. 5

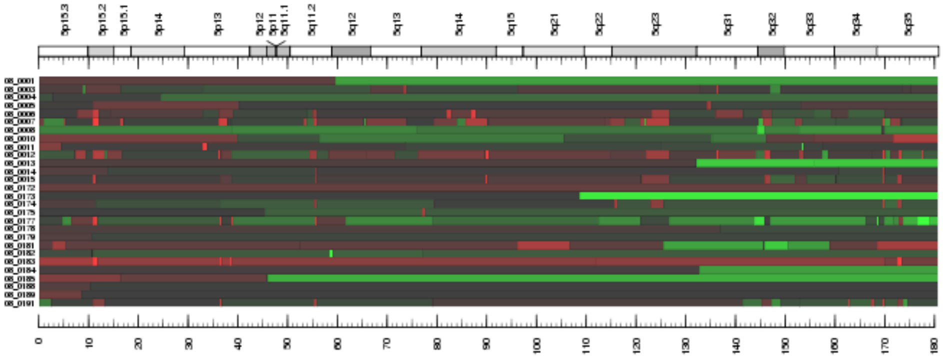

In-lab FFPE normals based reference

Chr. 6

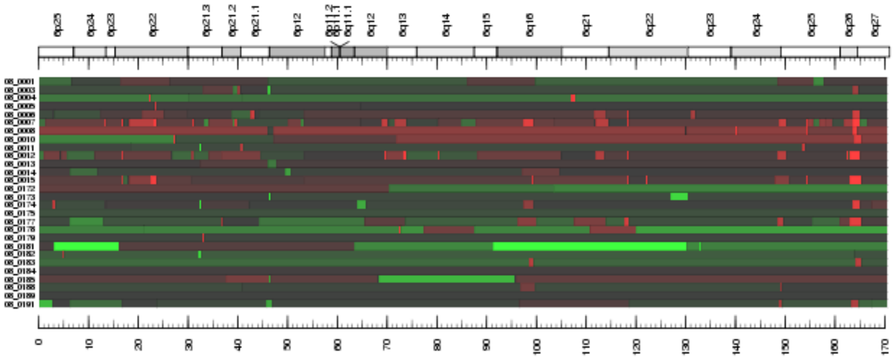

In-lab FFPE normals based reference

Chr. 7

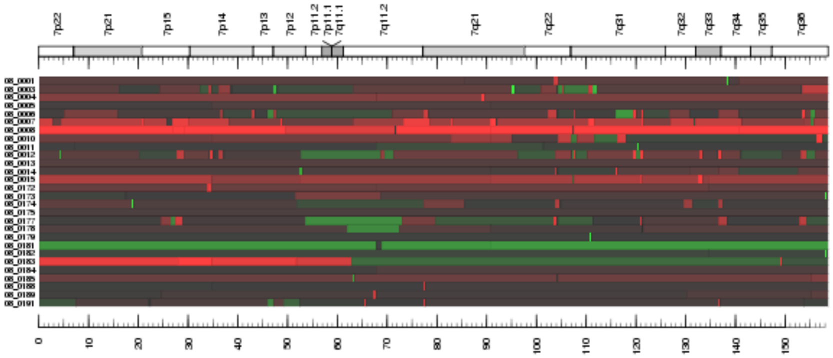

In-lab FFPE normals based reference

Chr. 8

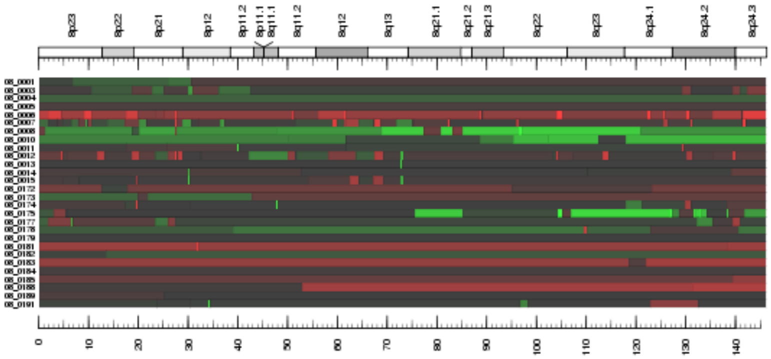

In-lab FFPE normals based reference

Chr. 9

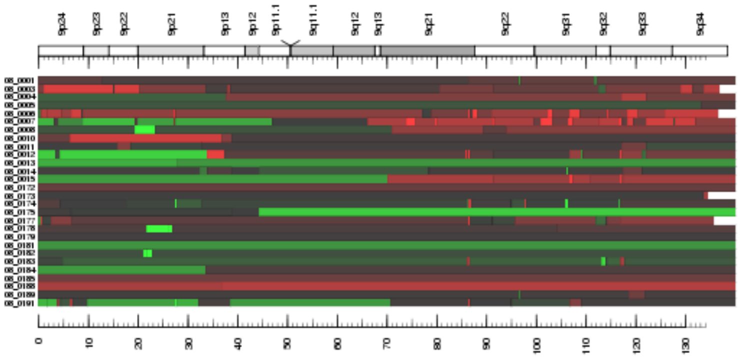

In-lab FFPE normals based reference

Chr. 10

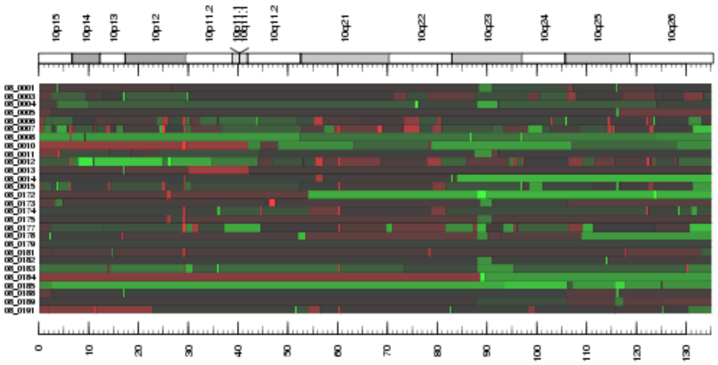

In-lab FFPE normals based reference

Chr. 11

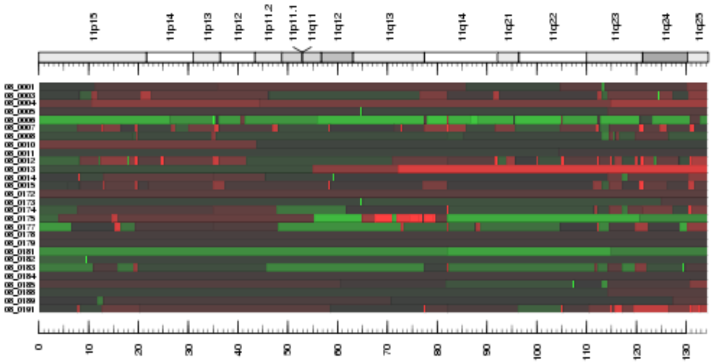

In-lab FFPE normals based reference

Chr. 12

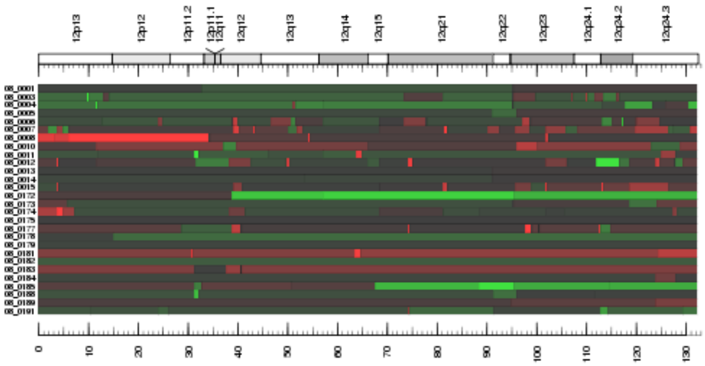

In-lab FFPE normals based reference

Chr. 13

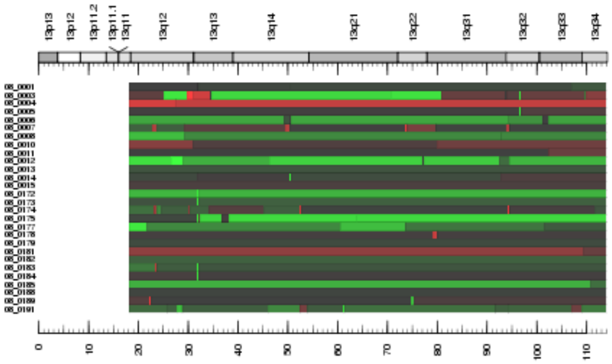

In-lab FFPE normals based reference

Chr. 14

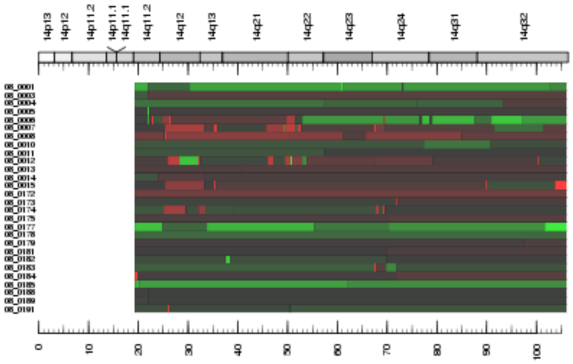

In-lab FFPE normals based reference

Chr. 15

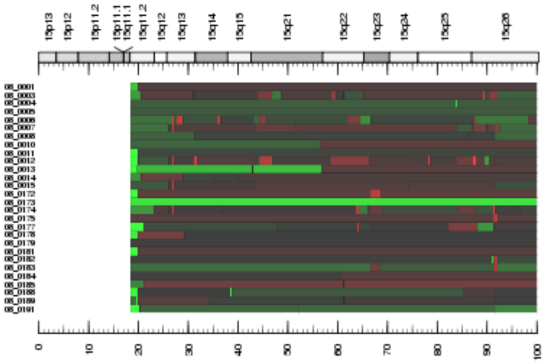

In-lab FFPE normals based reference

Chr. 16

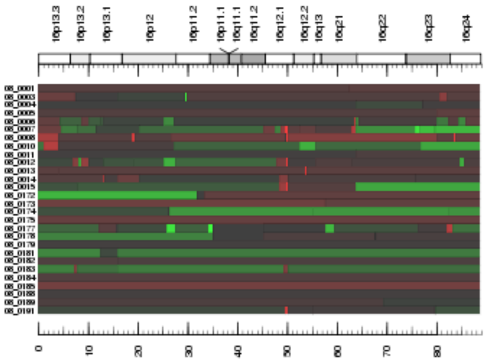

In-lab FFPE normals based reference

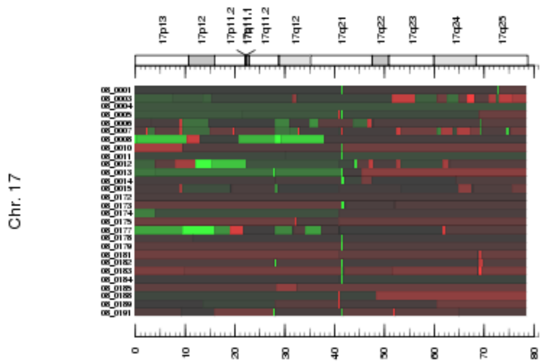

In-lab FFPE normals based reference

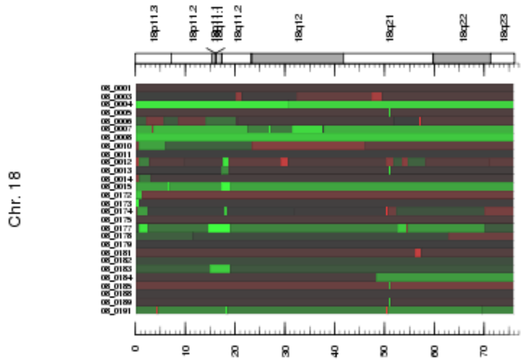

In-lab FFPE normals based reference

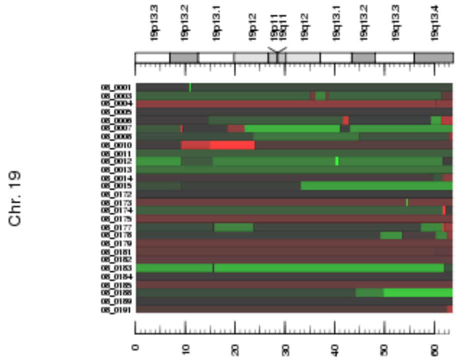

In-lab FFPE normals based reference

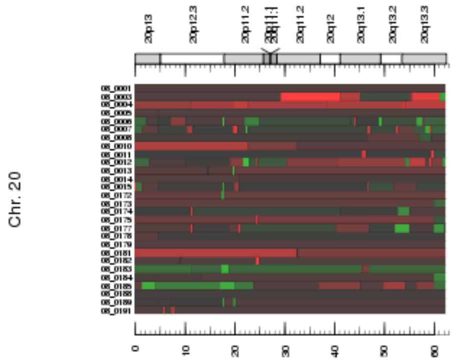

In-lab FFPE normals based reference

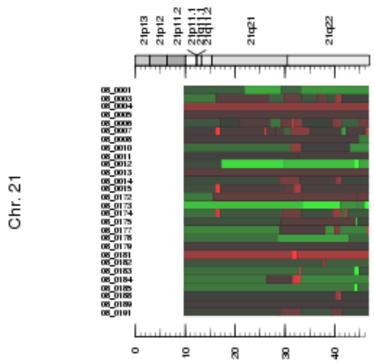

In-lab FFPE normals based reference

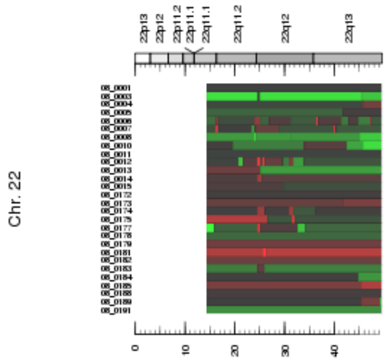

In-lab FFPE normals based reference

Figure S2

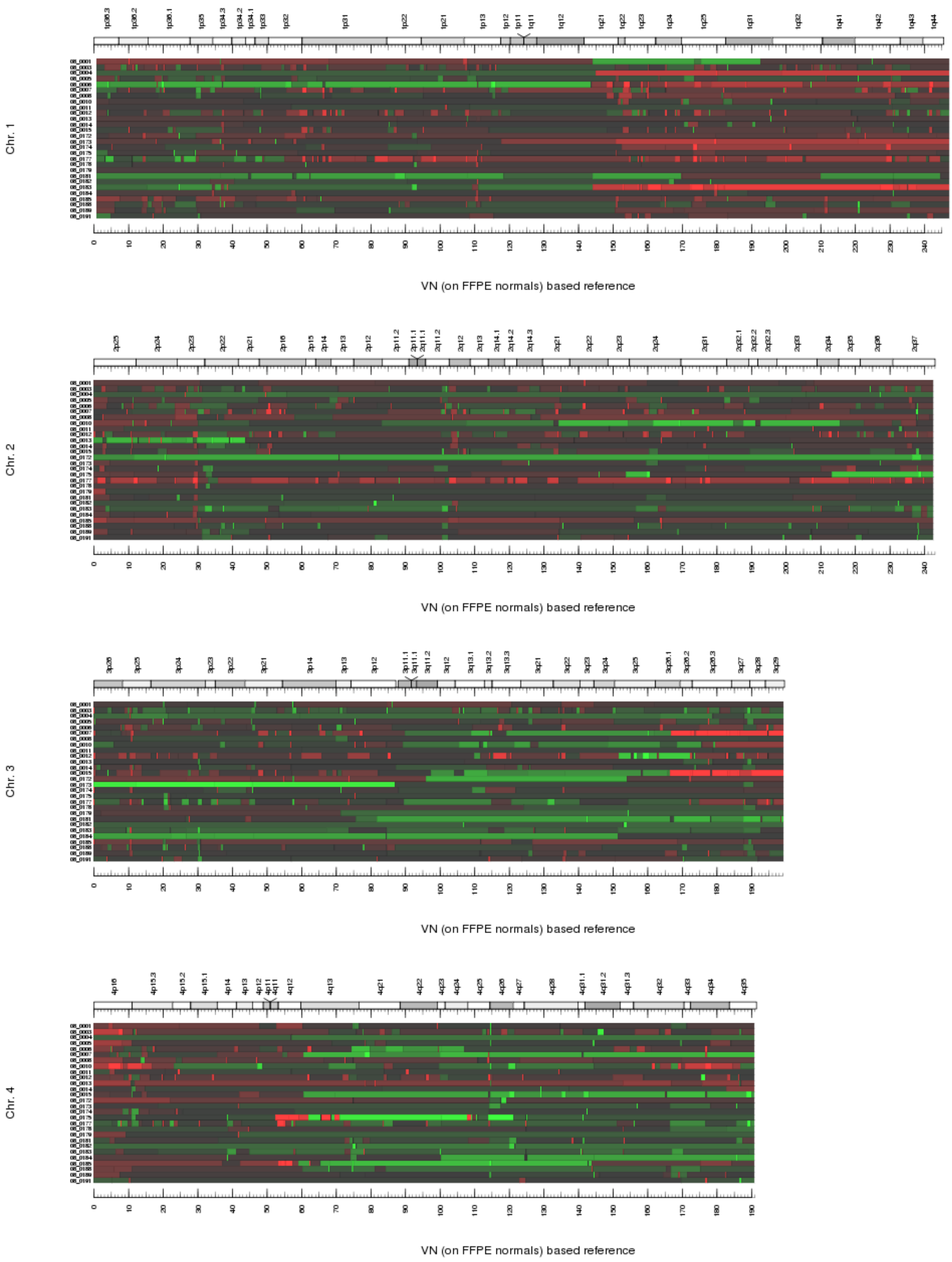
Chr. 1
VN (on FFPE normals) based reference
Chr. 2
VN (on FFPE normals) based reference
Chr. 3
VN (on FFPE normals) based reference
Chr. 4
VN (on FFPE normals) based reference

Chr. 5

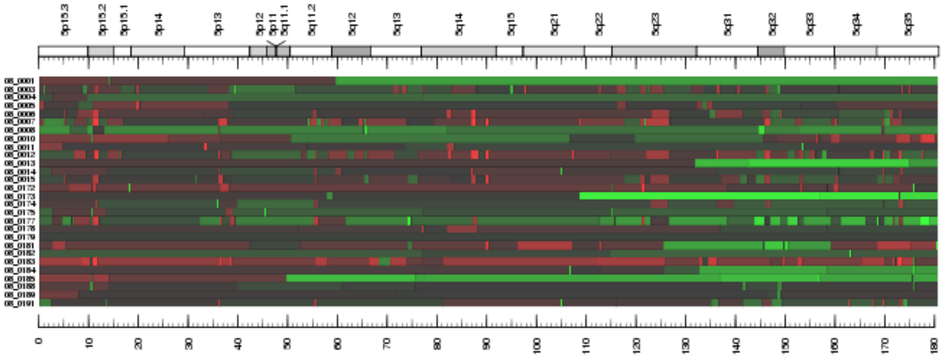

VN (on FFPE normals) based reference

Chr. 6

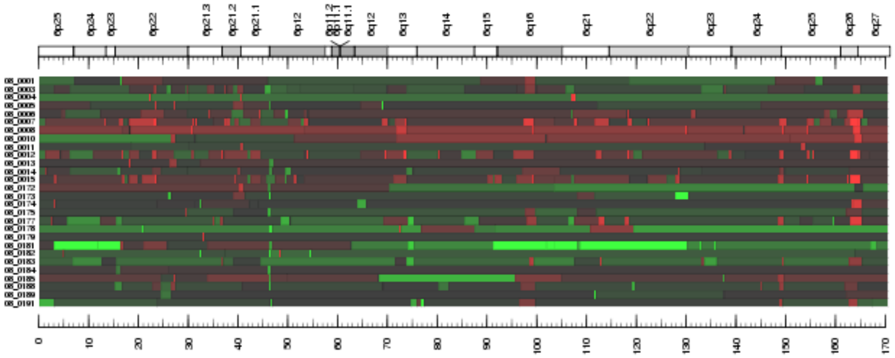

VN (on FFPE normals) based reference

Chr. 7

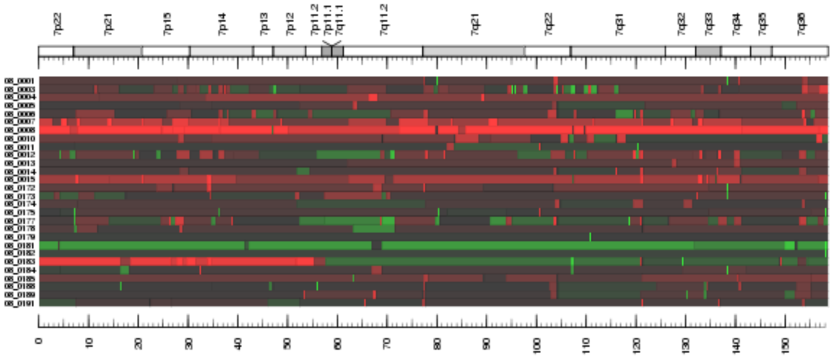

VN (on FFPE normals) based reference

Chr. 8

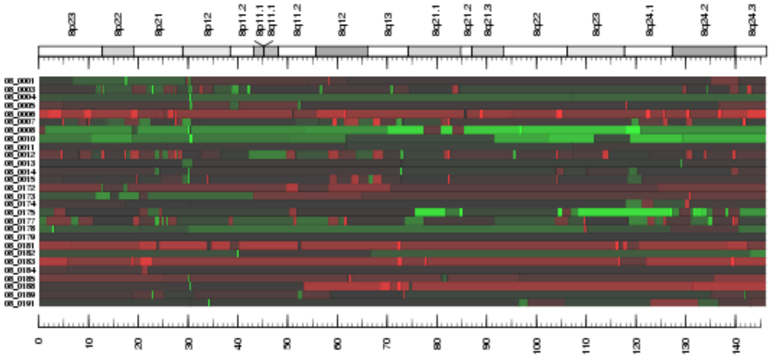

VN (on FFPE normals) based reference

Chr. 9

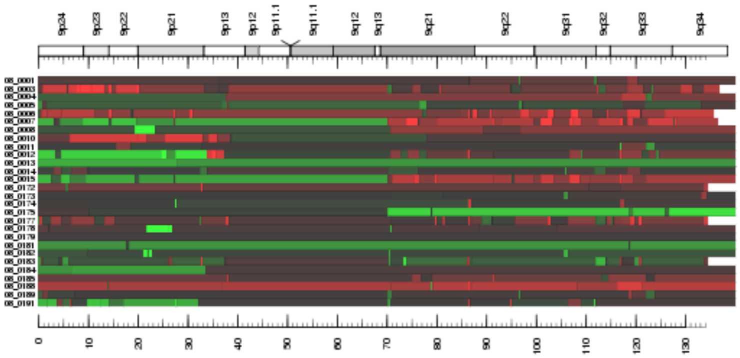

VN (on FFPE normals) based reference

Chr. 10

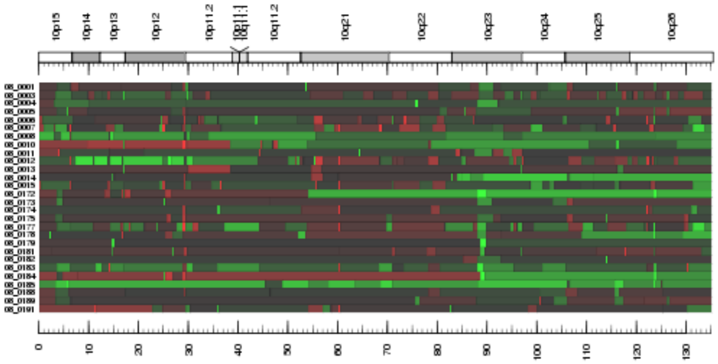

VN (on FFPE normals) based reference

Chr. 11

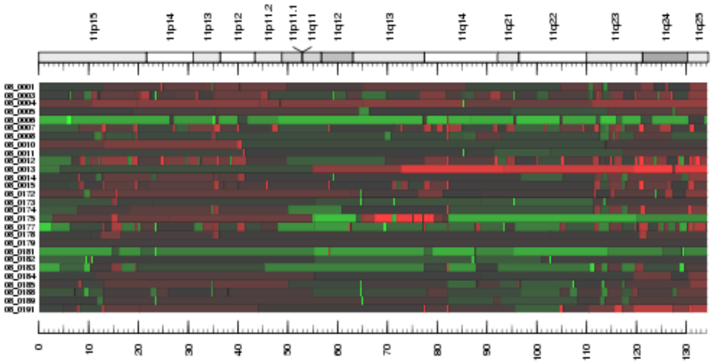

VN (on FFPE normals) based reference

Chr. 12

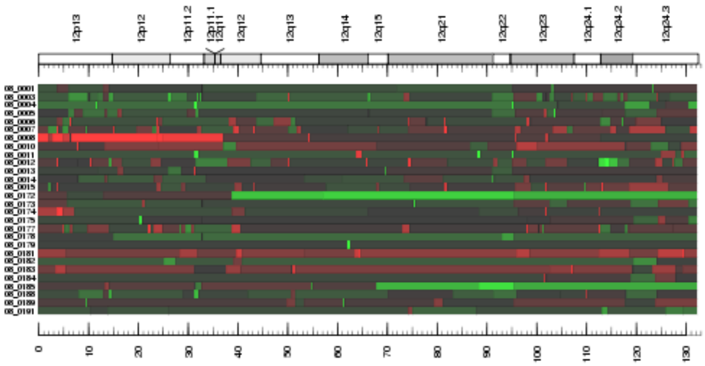

VN (on FFPE normals) based reference

Chr. 13

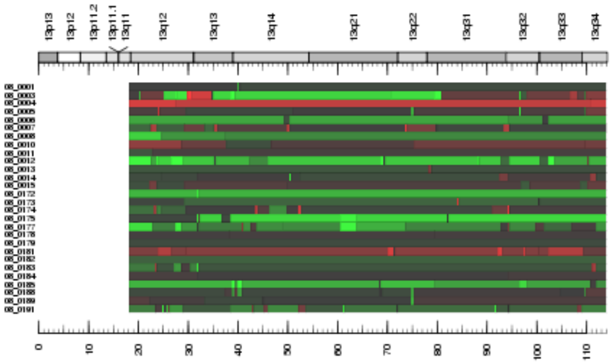

VN (on FFPE normals) based reference

Chr. 14

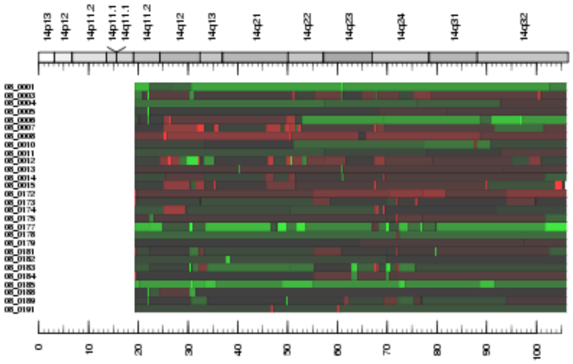

VN (on FFPE normals) based reference

Chr. 15

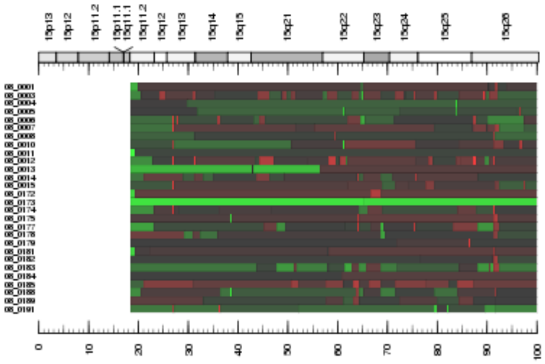

VN (on FFPE normals) based reference

Chr. 16

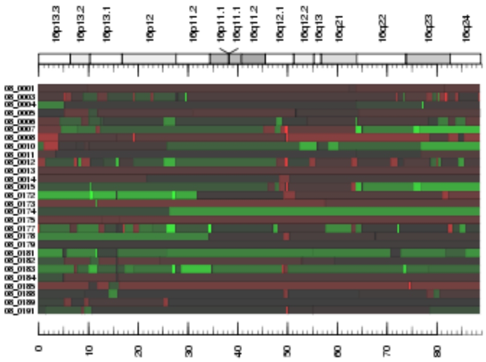

VN (on FFPE normals) based reference

Chr. 17

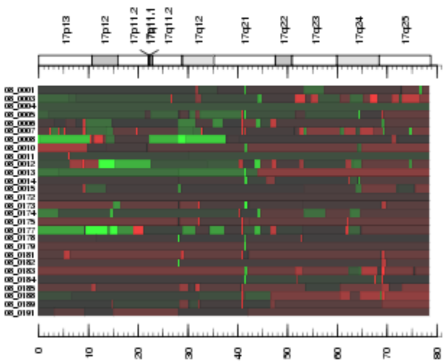

VN (on FFPE normals) based reference

Chr. 18

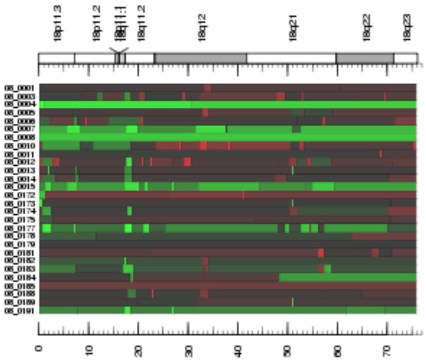

VN (on FFPE normals) based reference

Chr. 19

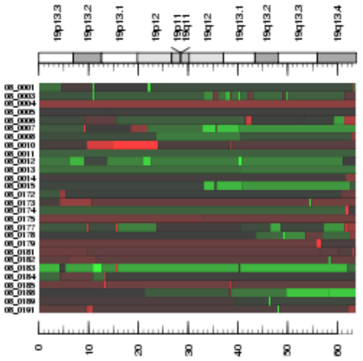

VN (on FFPE normals) based reference

Chr. 20

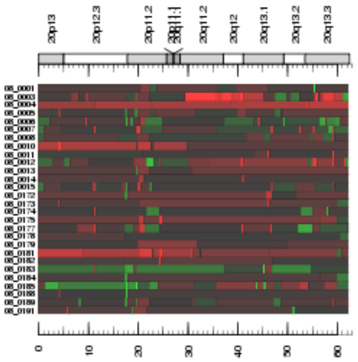

VN (on FFPE normals) based reference

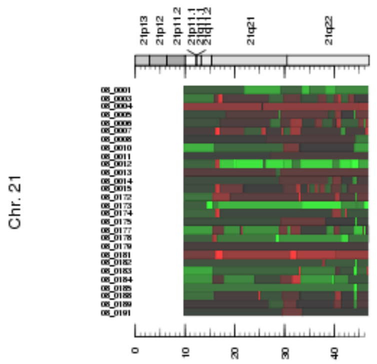

VN (on FFPE normals) based reference

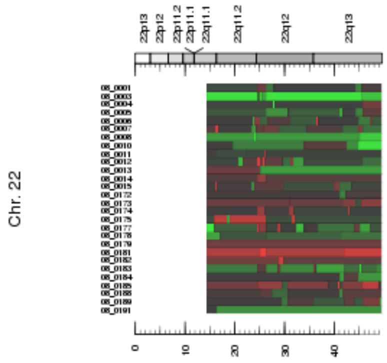

VN (on FFPE normals) based reference

Figure S3

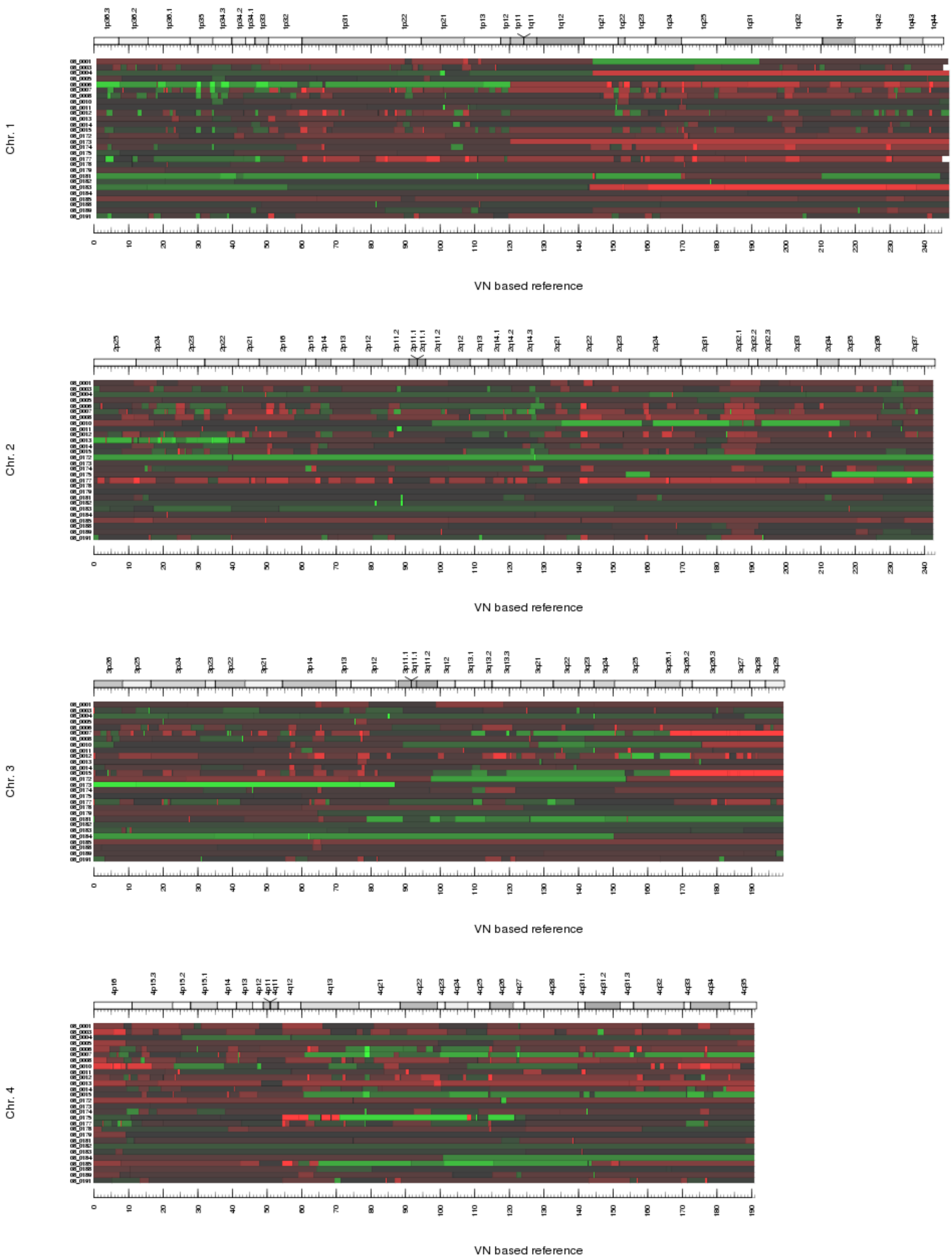
Chr. 1
VN based reference
Chr. 2
VN based reference
Chr. 3
VN based reference
Chr. 4
VN based reference

Chr. 5

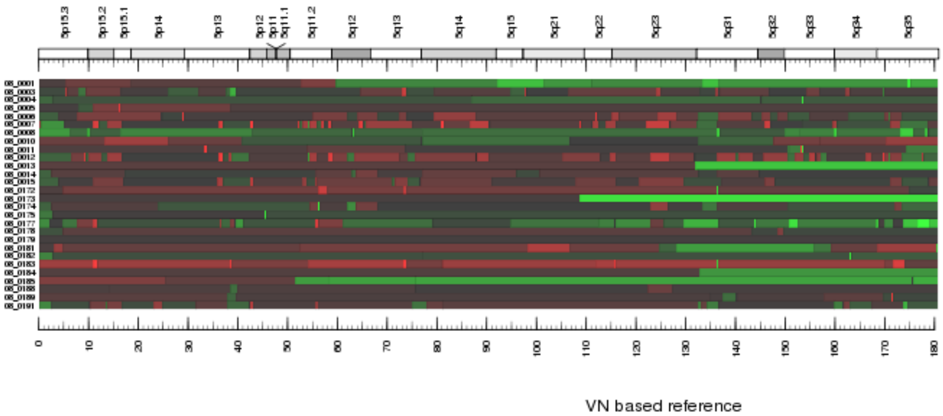

Chr. 6

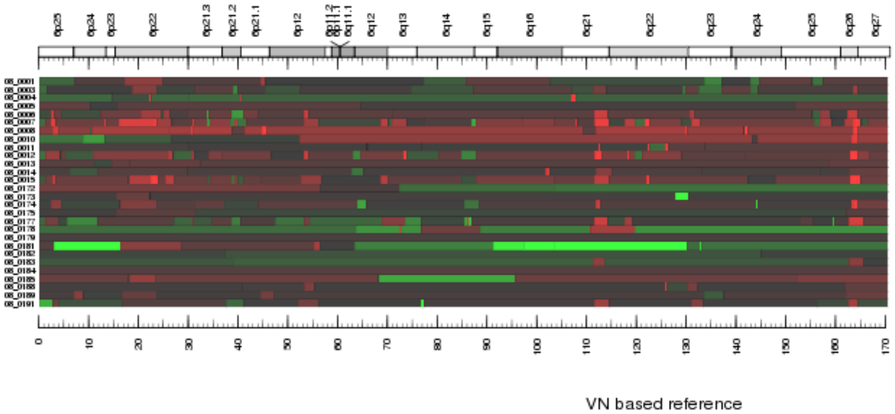

Chr. 7

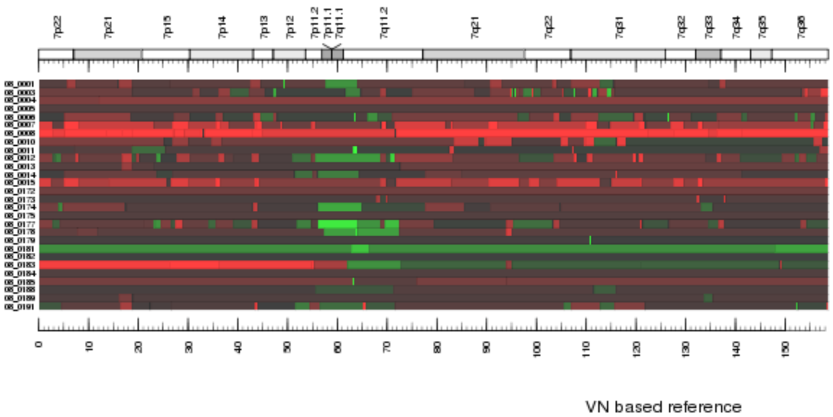

Chr. 8

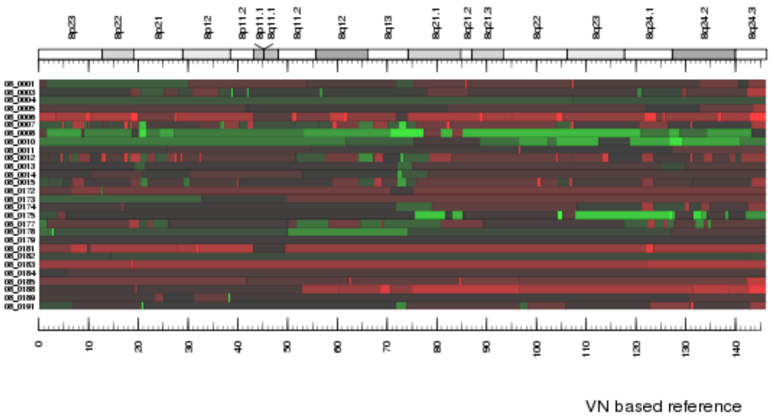

Chr. 9

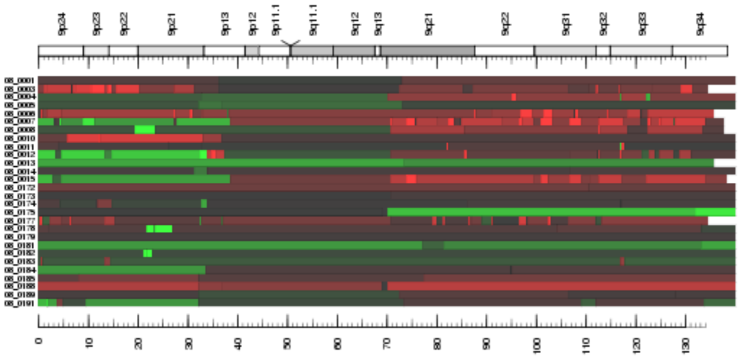

Chr. 10

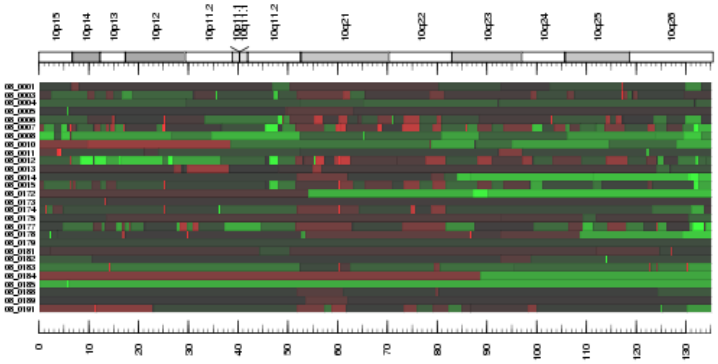

Chr. 11

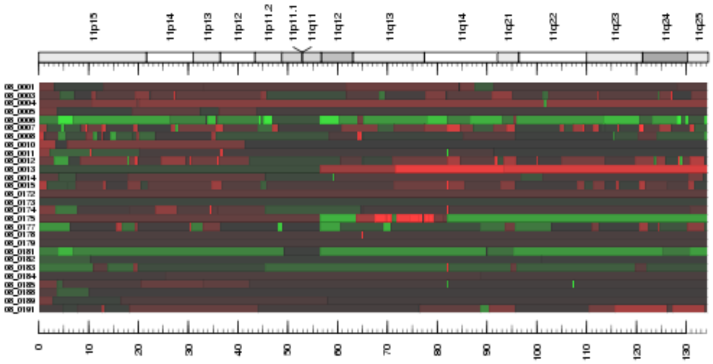

Chr. 12

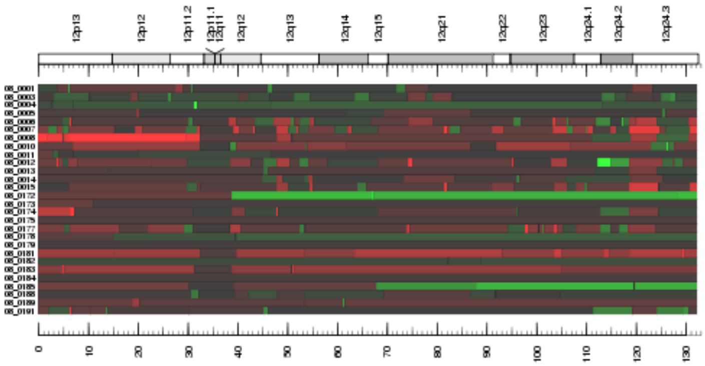

Chr. 13

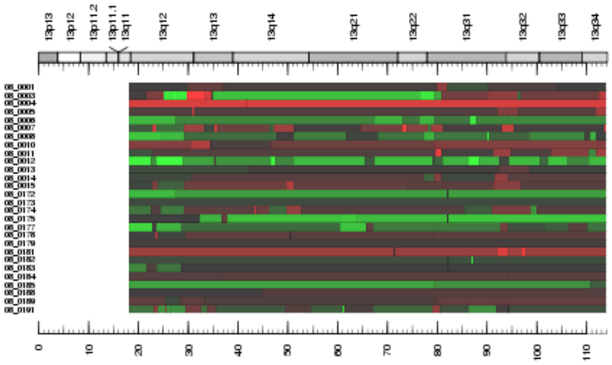

VN based reference

Chr. 14

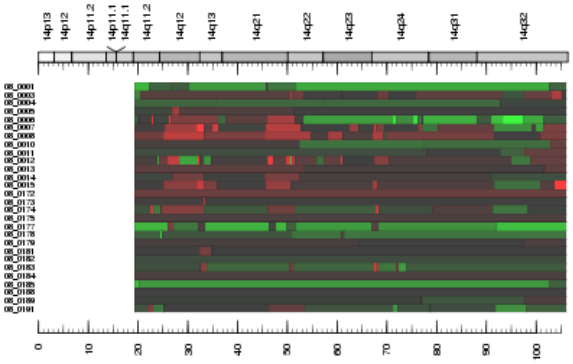

VN based reference

Chr. 15

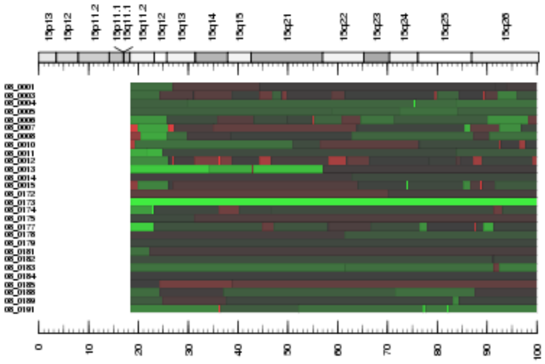

VN based reference

Chr. 16

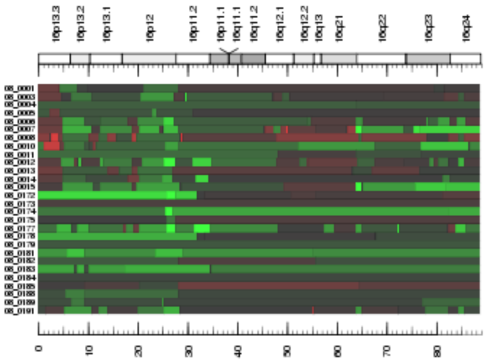

VN based reference

Chr. 17

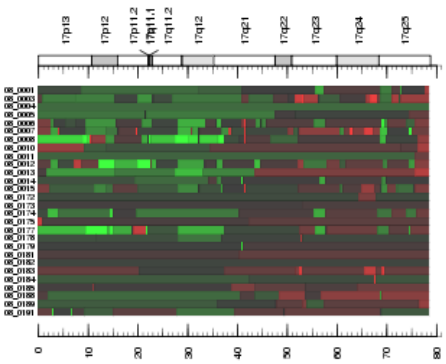

VN based reference

Chr. 18

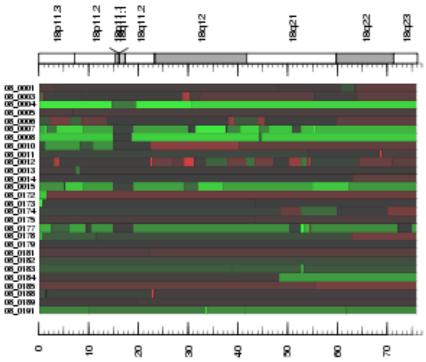

VN based reference

Chr. 19

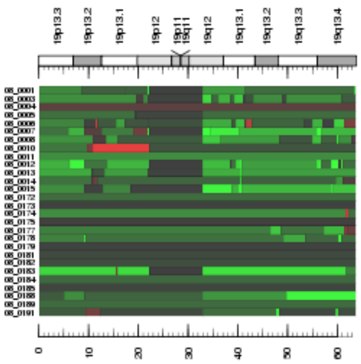

VN based reference

Chr. 20

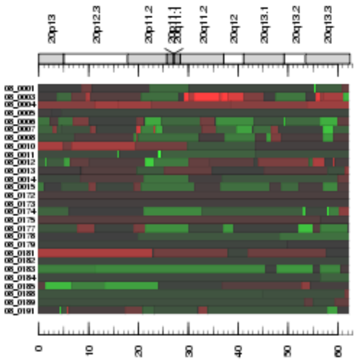

VN based reference

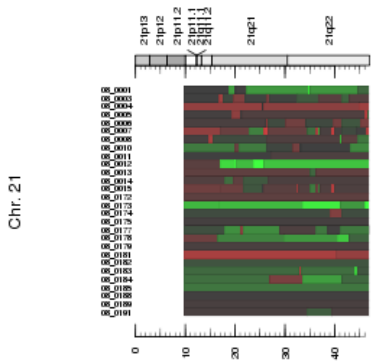

VN based reference

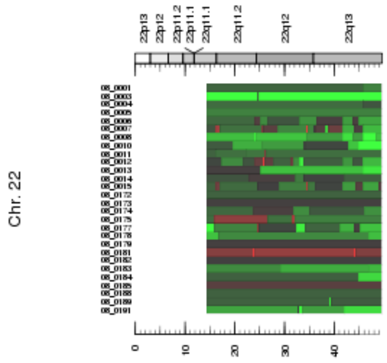

VN based reference

Figure S4

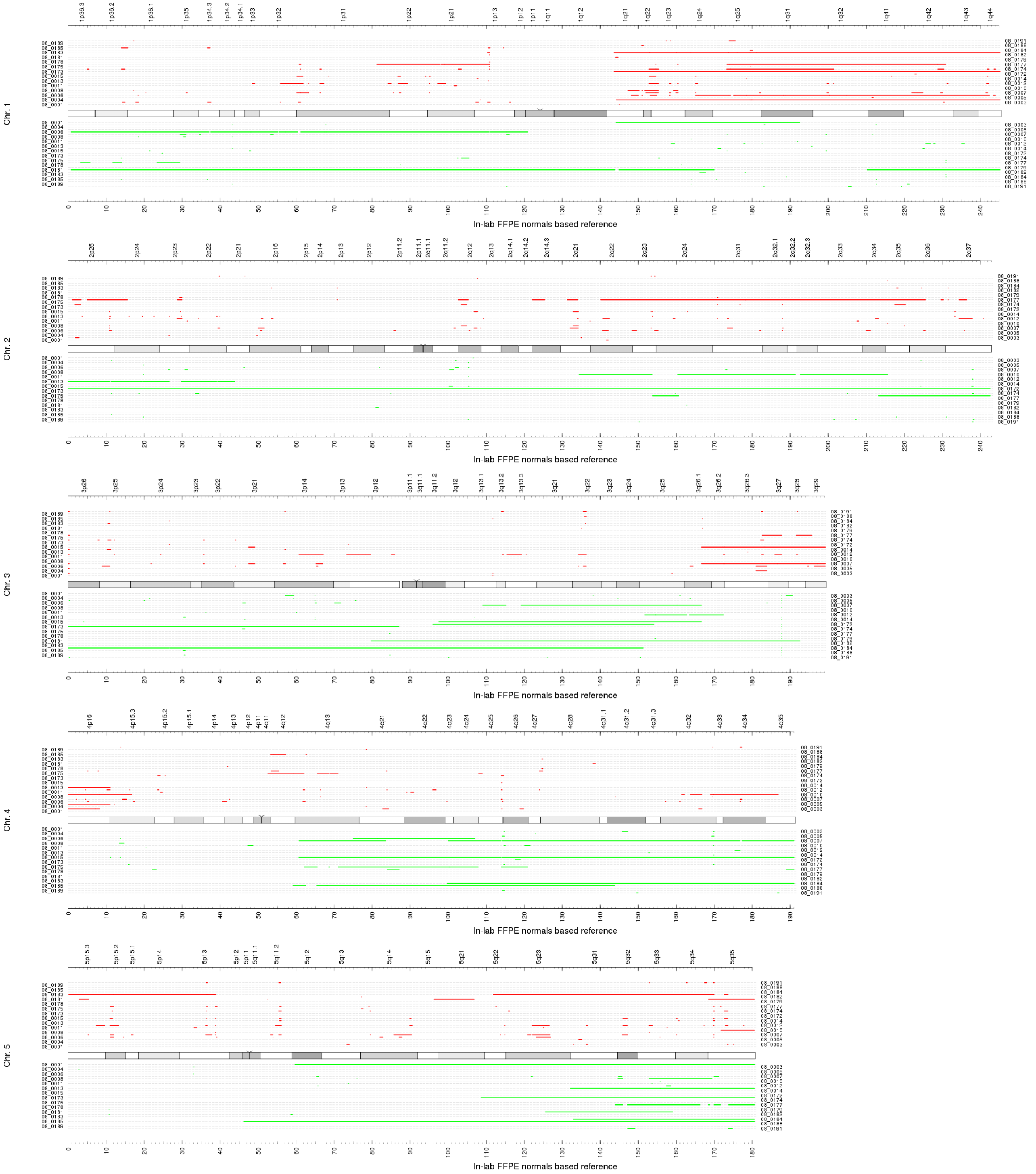

Chr. 1
In-lab FFPE normals based reference
Chr. 2
In-lab FFPE normals based reference
Chr. 3
In-lab FFPE normals based reference
Chr. 4
In-lab FFPE normals based reference
Chr. 5
In-lab FFPE normals based reference

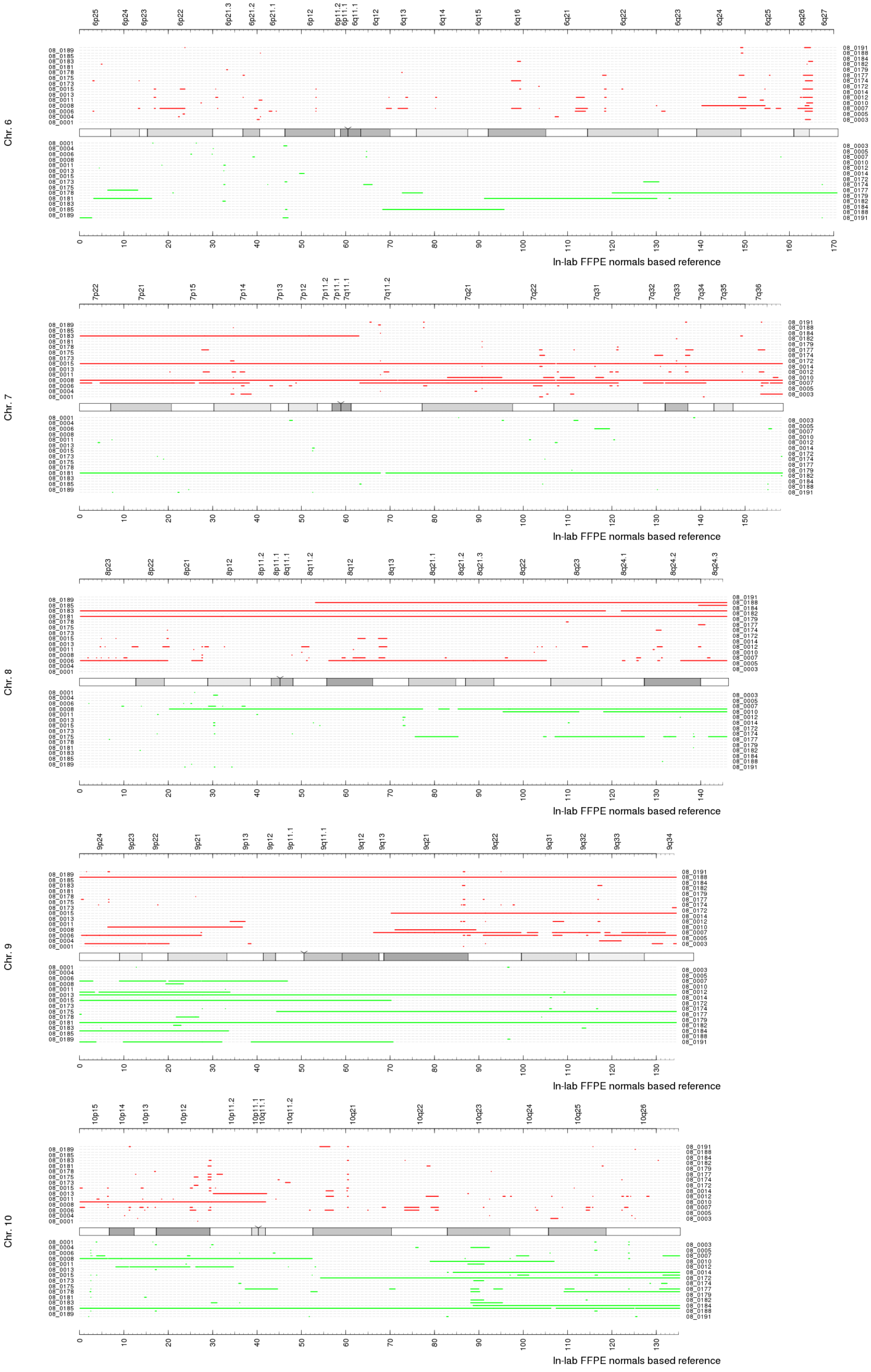

Chr. 6
In-lab FFPE normals based reference
Chr. 7
In-lab FFPE normals based reference
Chr. 8
In-lab FFPE normals based reference
Chr. 9
In-lab FFPE normals based reference
Chr. 10
In-lab FFPE normals based reference

Chr. 11

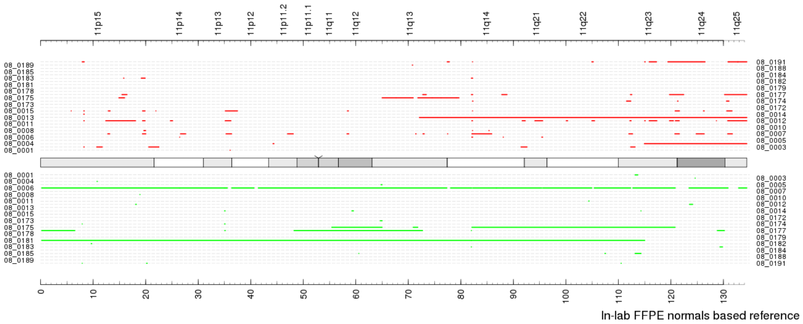

Chr. 12

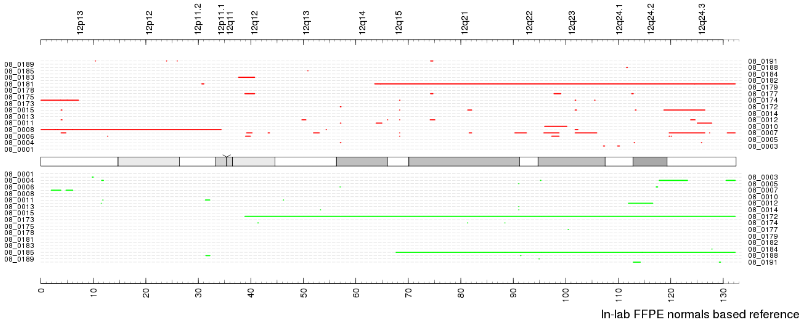

Chr. 13

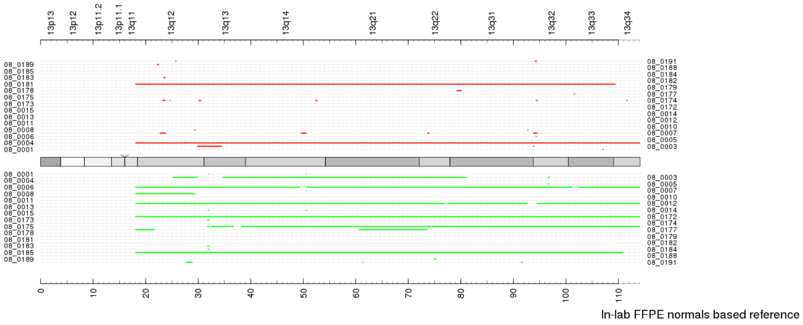

Chr. 14

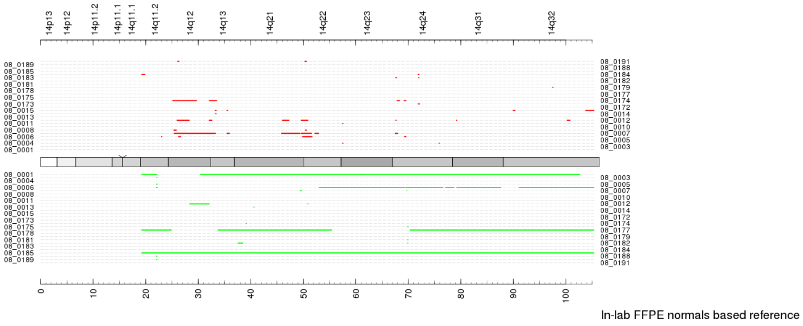

Chr. 15

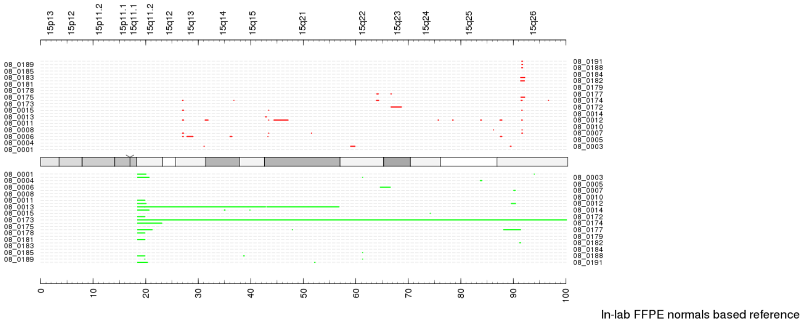

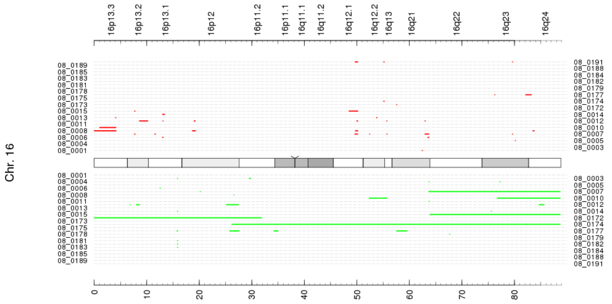

In-lab FFPE normals based reference

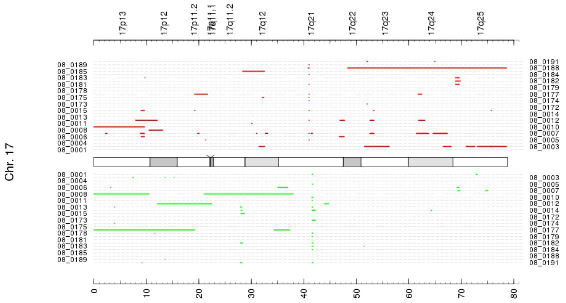

In-lab FFPE normals based reference

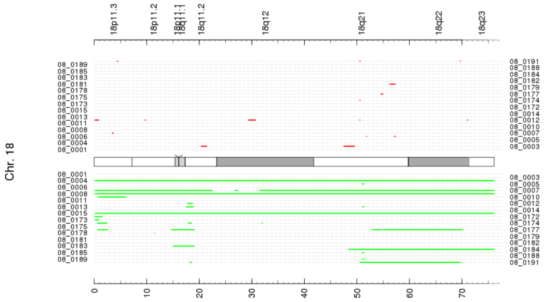

In-lab FFPE normals based reference

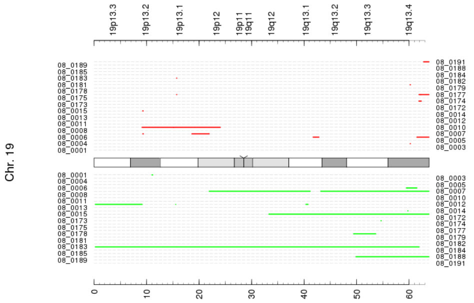

In-lab FFPE normals based reference

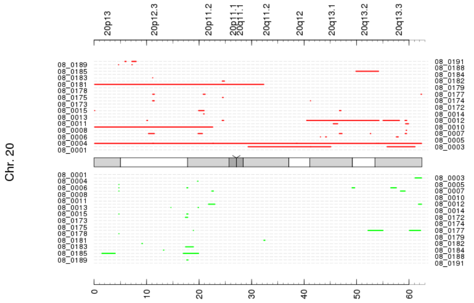

In-lab FFPE normals based reference

Chr. 21

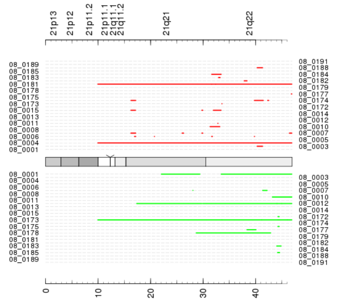

In-lab FFPE normals based reference

Chr. 22

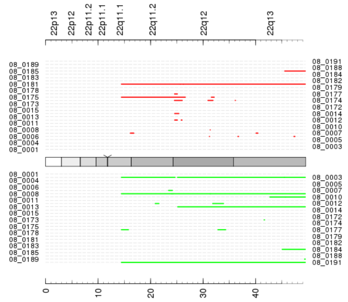

In-lab FFPE normals based reference

Figure S5

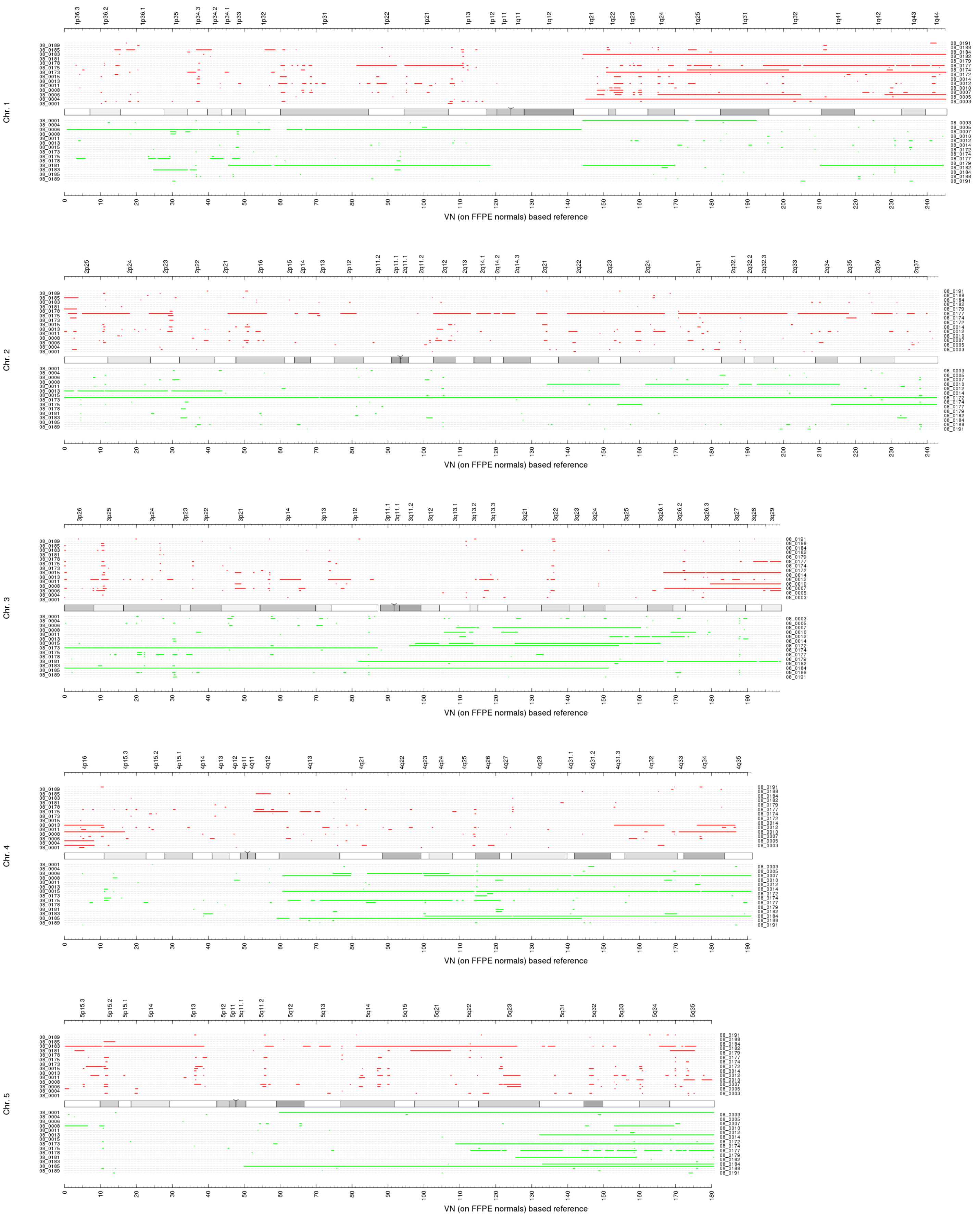

Chr. 1
VN (on FFPE normals) based reference
Chr. 2
VN (on FFPE normals) based reference
Chr. 3
VN (on FFPE normals) based reference
Chr. 4
VN (on FFPE normals) based reference
Chr. 5
VN (on FFPE normals) based reference

Chr. 6

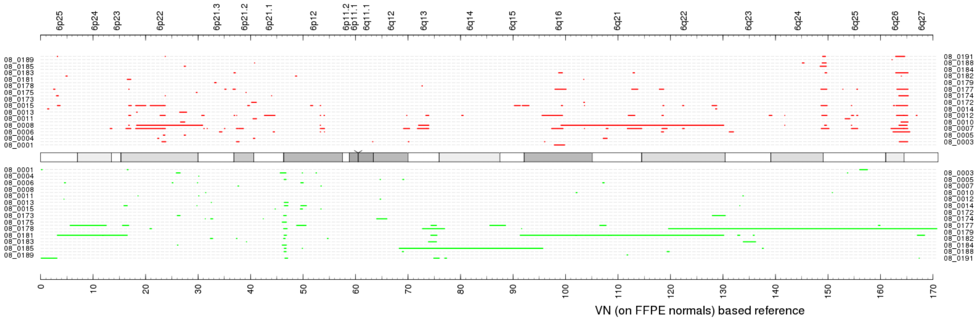

Chr. 7

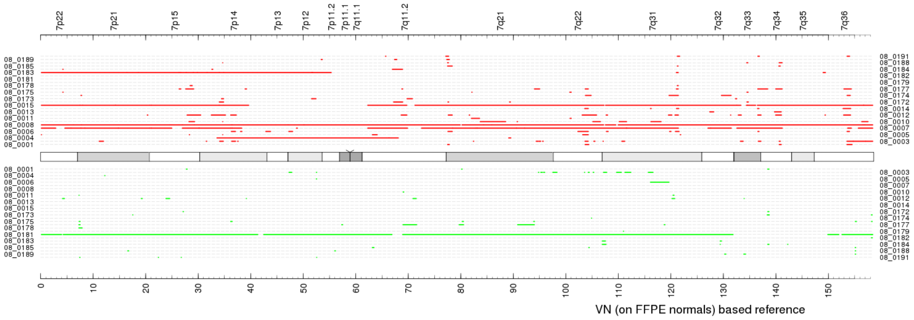

Chr. 8

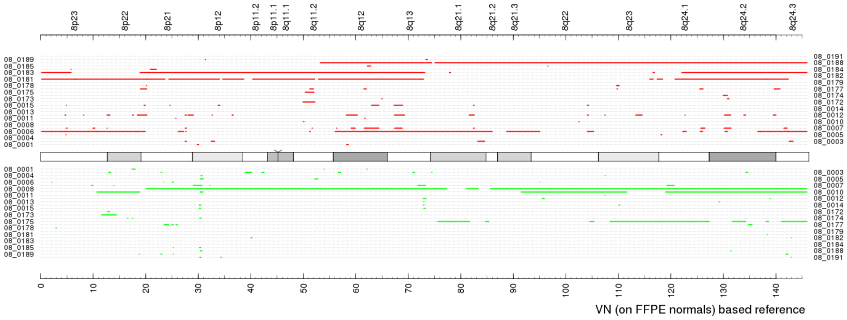

Chr. 9

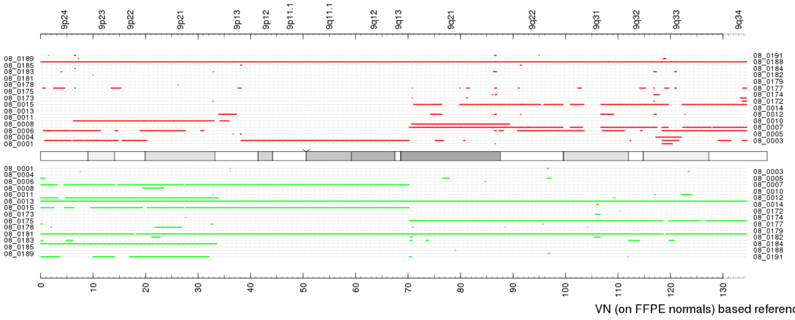

Chr. 10

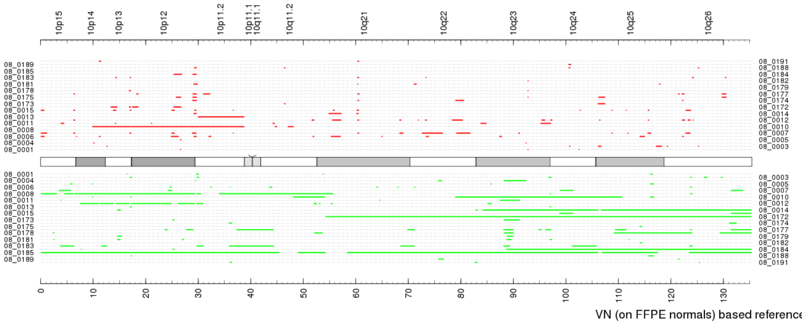

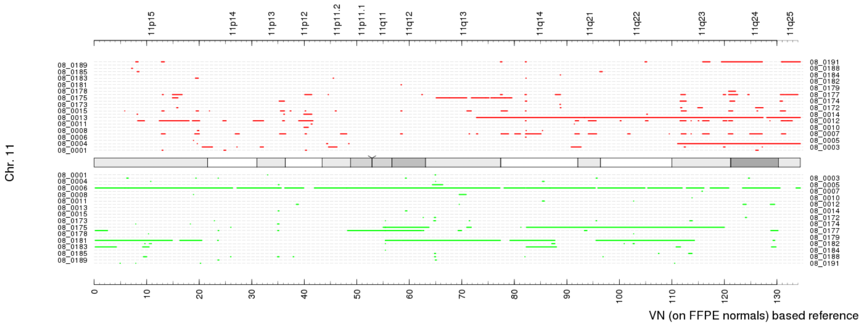
Chr. 11
11p15
11p14
11p13
11p12
11p11.2
11p11.1
11q11
11q12
11q13
11q14
11q21
11q22
11q23
11q24
11q25
VN (on FFPE normals) based reference

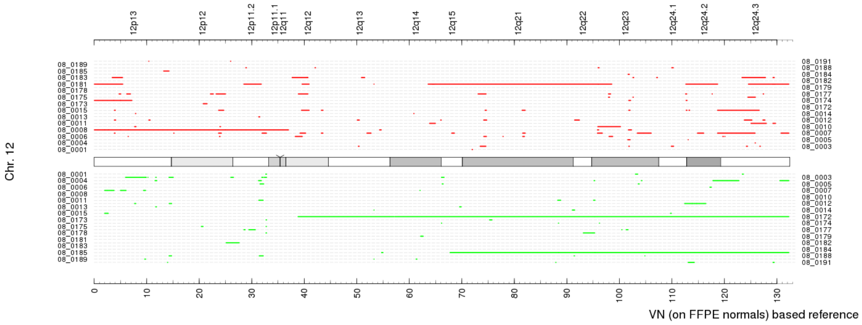
Chr. 12
12p13
12p12
12p11.2
12p11.1
12q11
12q12
12q13
12q14
12q15
12q21
12q22
12q23
12q24.1
12q24.2
12q24.3
VN (on FFPE normals) based reference

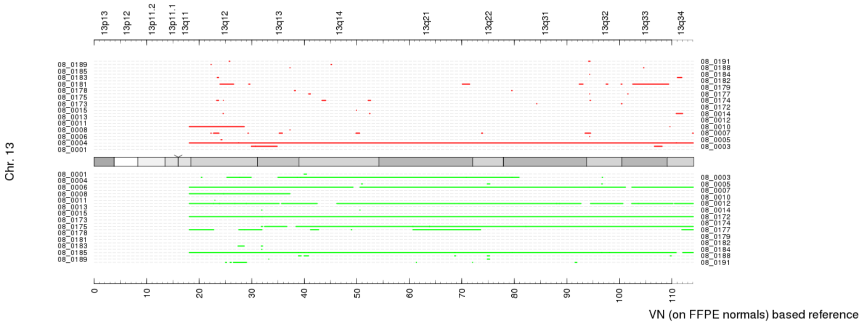
Chr. 13
13p13
13p12
13p11.2
13p11.1
13q11
13q12
13q13
13q14
13q21
13q22
13q31
13q32
13q33
13q34
VN (on FFPE normals) based reference

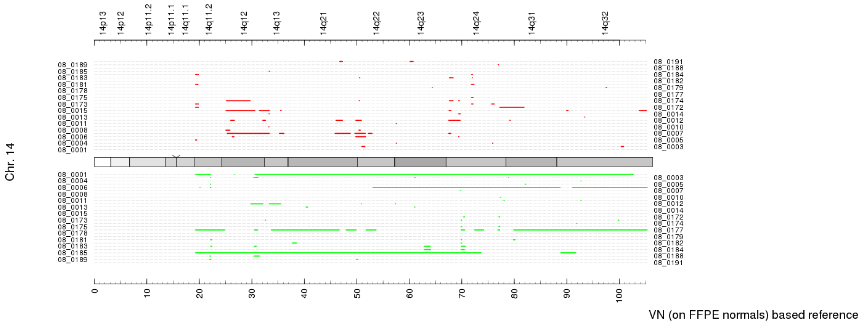
Chr. 14
14p13
14p12
14p11.2
14p11.1
14q11.1
14q11.2
14q12
14q13
14q21
14q22
14q23
14q24
14q31
14q32
VN (on FFPE normals) based reference

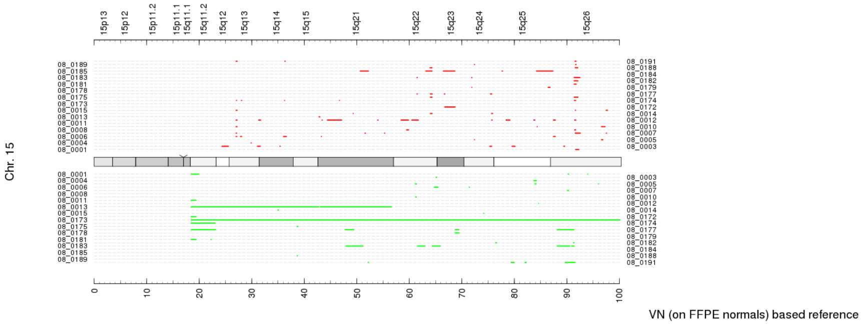
Chr. 15
15p13
15p12
15p11.2
15p11.1
15q11.1
15q11.2
15q12
15q13
15q14
15q15
15q21
15q22
15q23
15q24
15q25
15q26
VN (on FFPE normals) based reference

Chr. 16

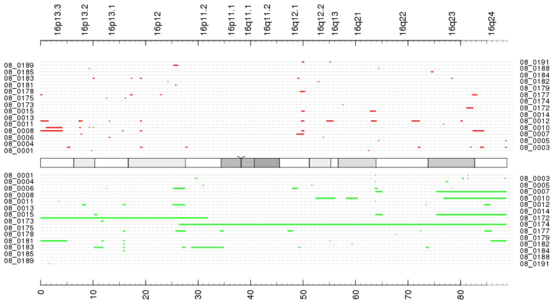

VN (on FFPE normals) based reference

Chr. 17

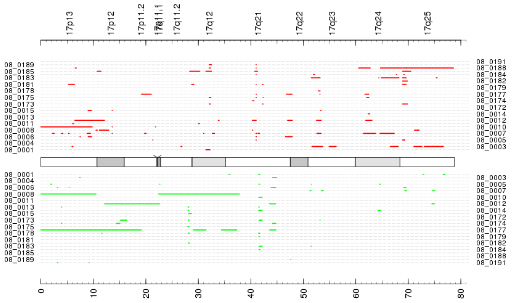

VN (on FFPE normals) based reference

Chr. 18

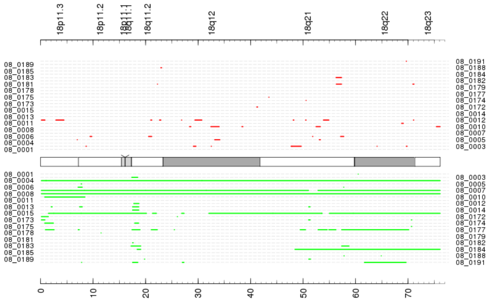

VN (on FFPE normals) based reference

Chr. 19

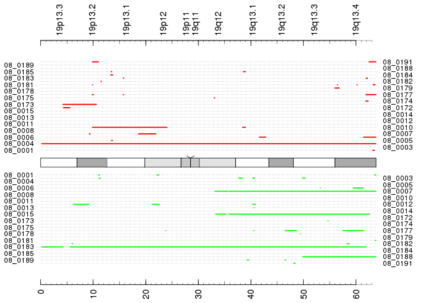

VN (on FFPE normals) based reference

Chr. 20

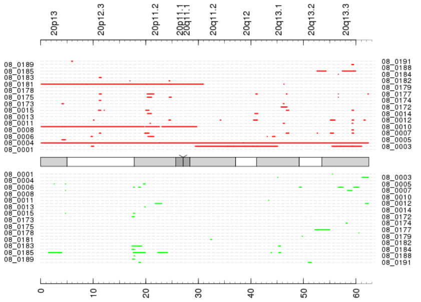

VN (on FFPE normals) based reference

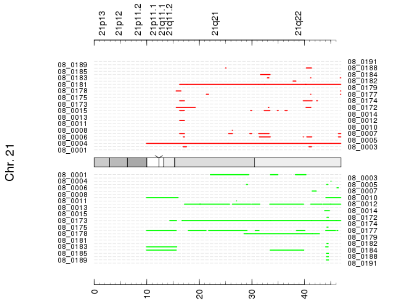

VN (on FFPE normals) based reference

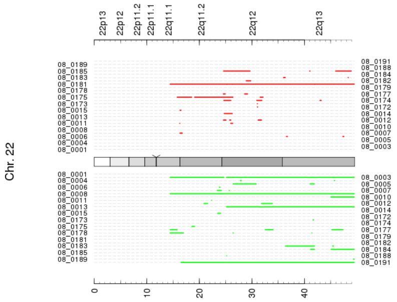

VN (on FFPE normals) based reference

Figure S6

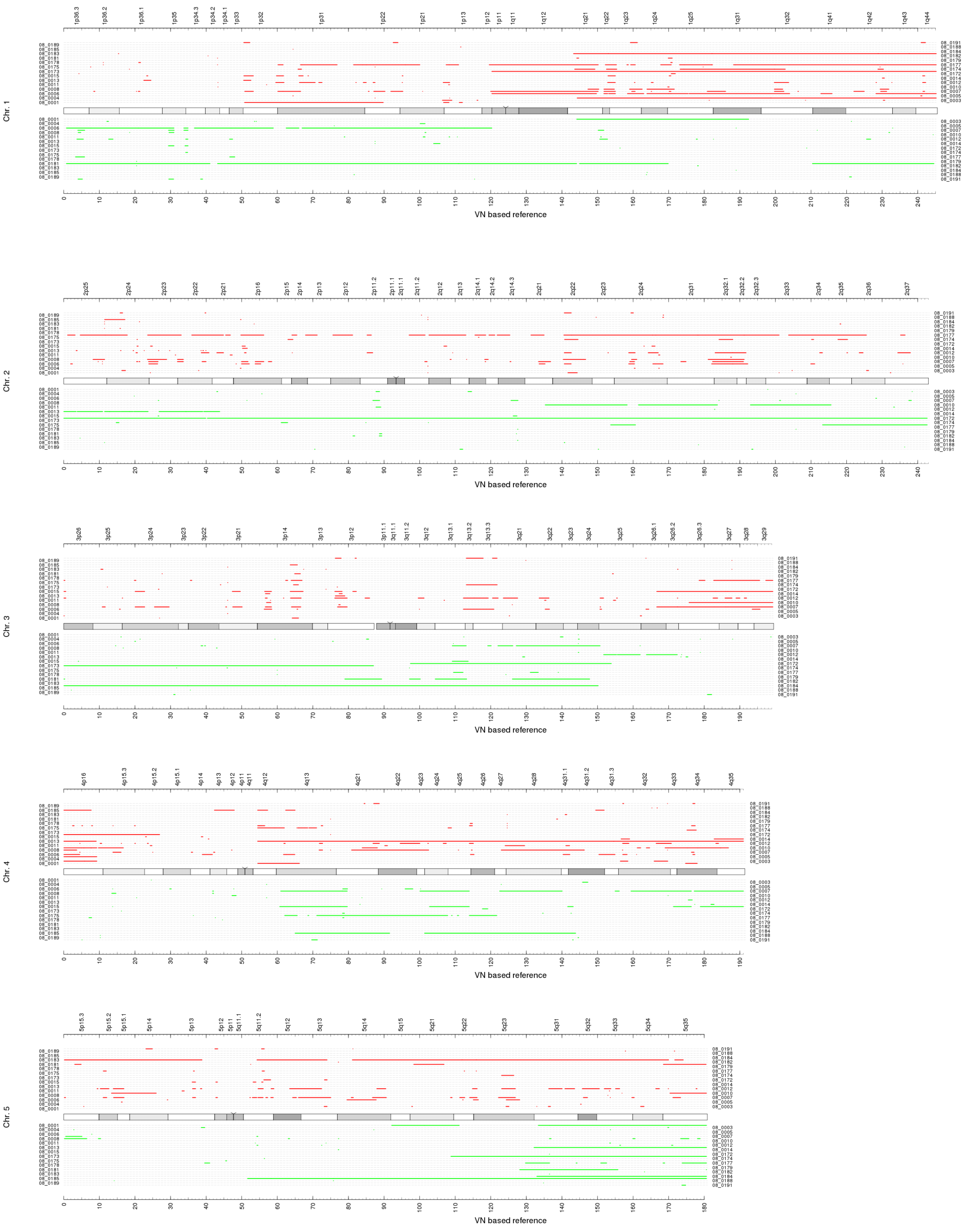

Chr. 6

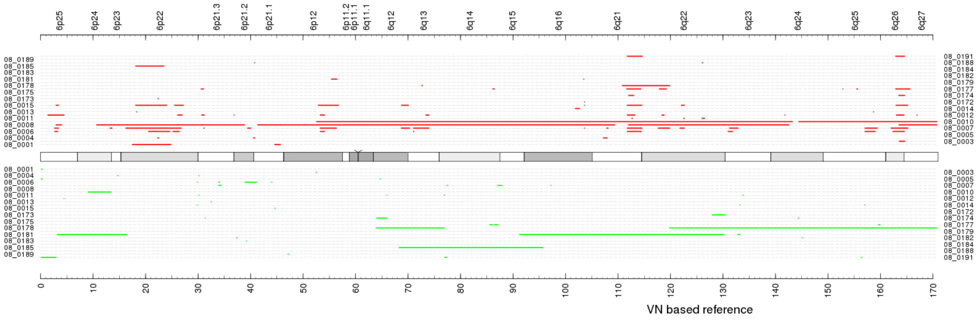

Chr. 7

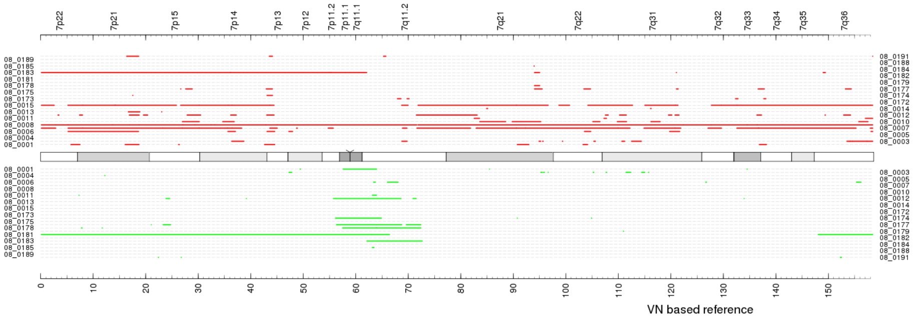

Chr. 8

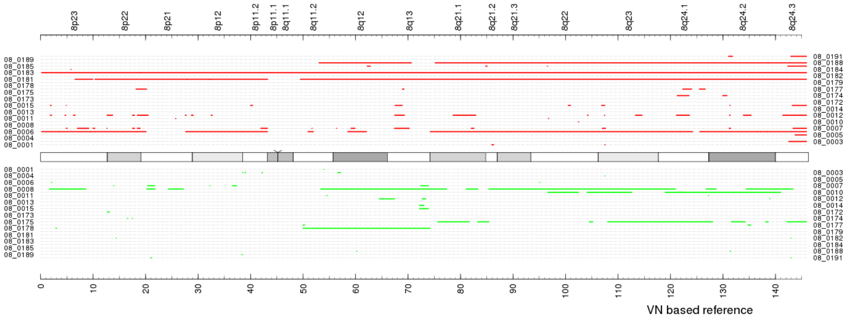

Chr. 9

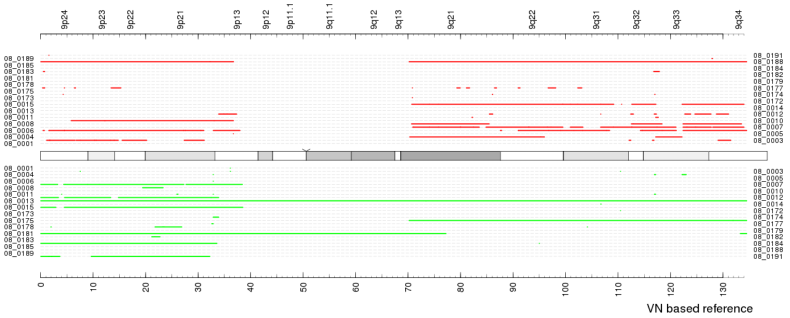

Chr. 10

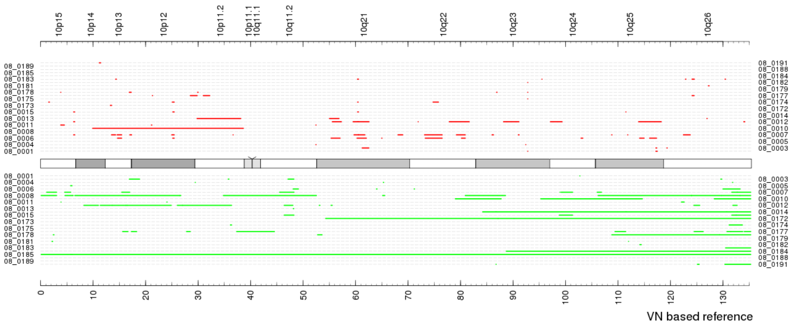

Chr. 11

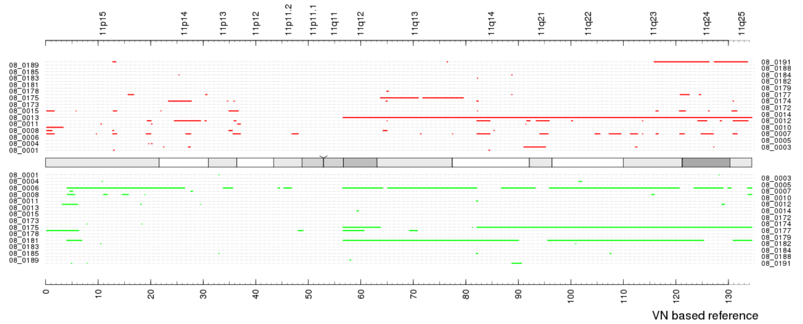

Chr. 12

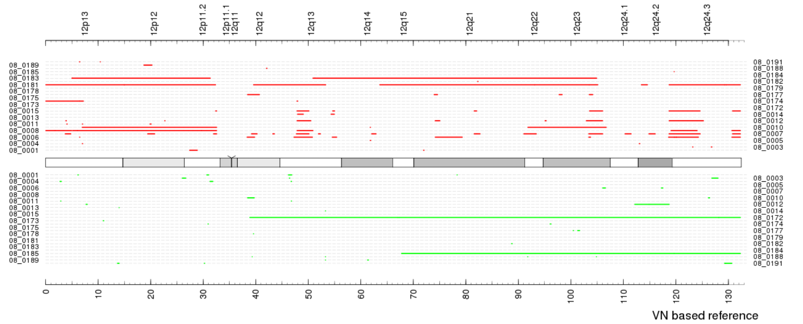

Chr. 13

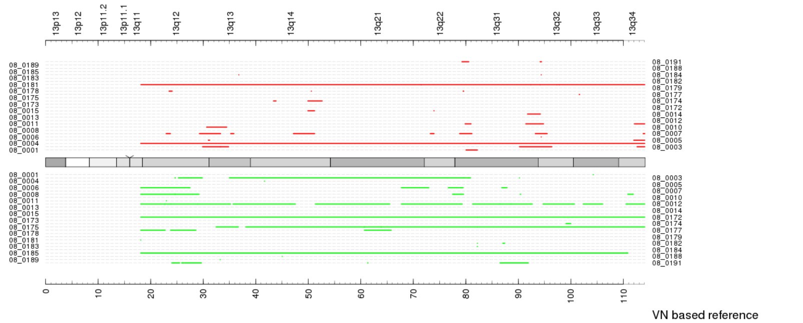

Chr. 14

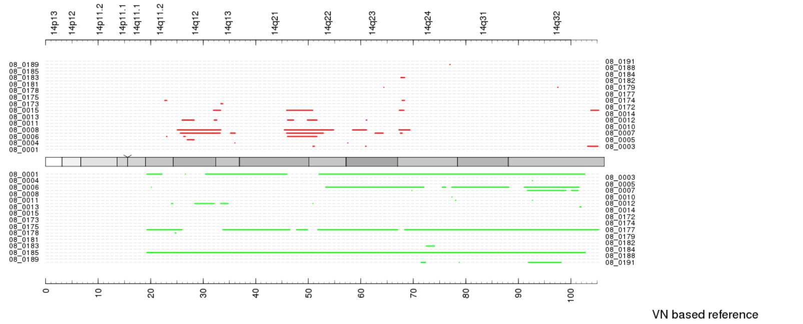

Chr. 15

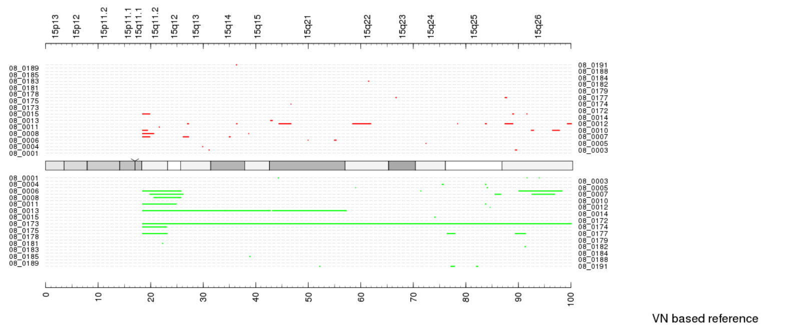

Chr. 16

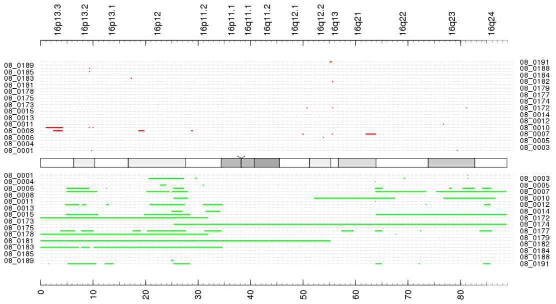

VN based reference

Chr. 17

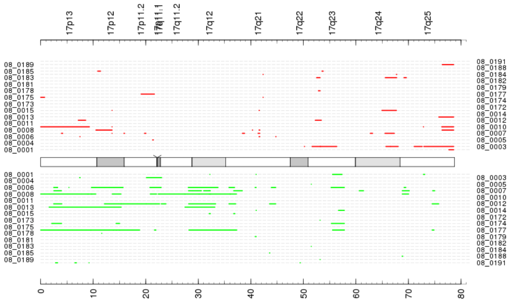

VN based reference

Chr. 18

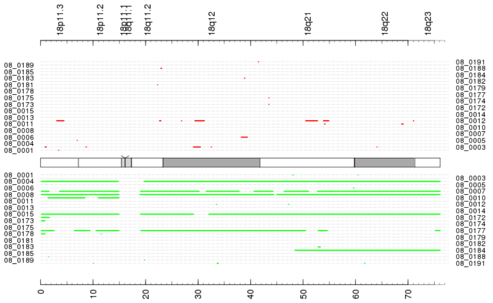

VN based reference

Chr. 19

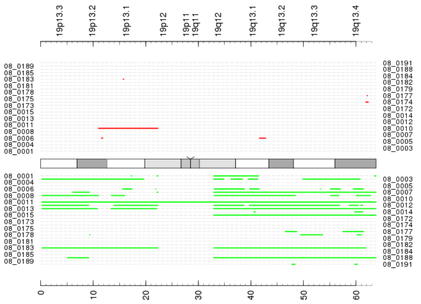

VN based reference

Chr. 20

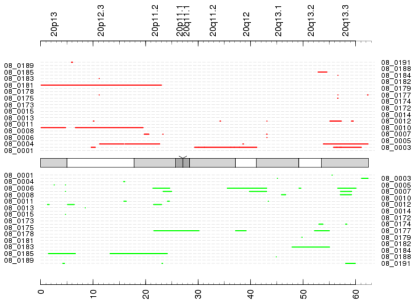

VN based reference

Chr. 21

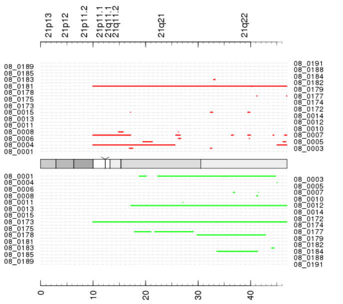

VN based reference

Chr. 22

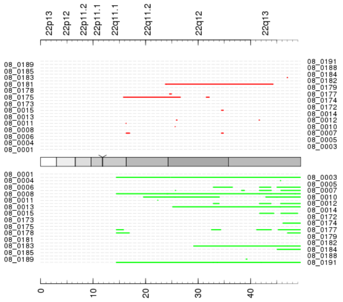

VN based reference

Figure S7

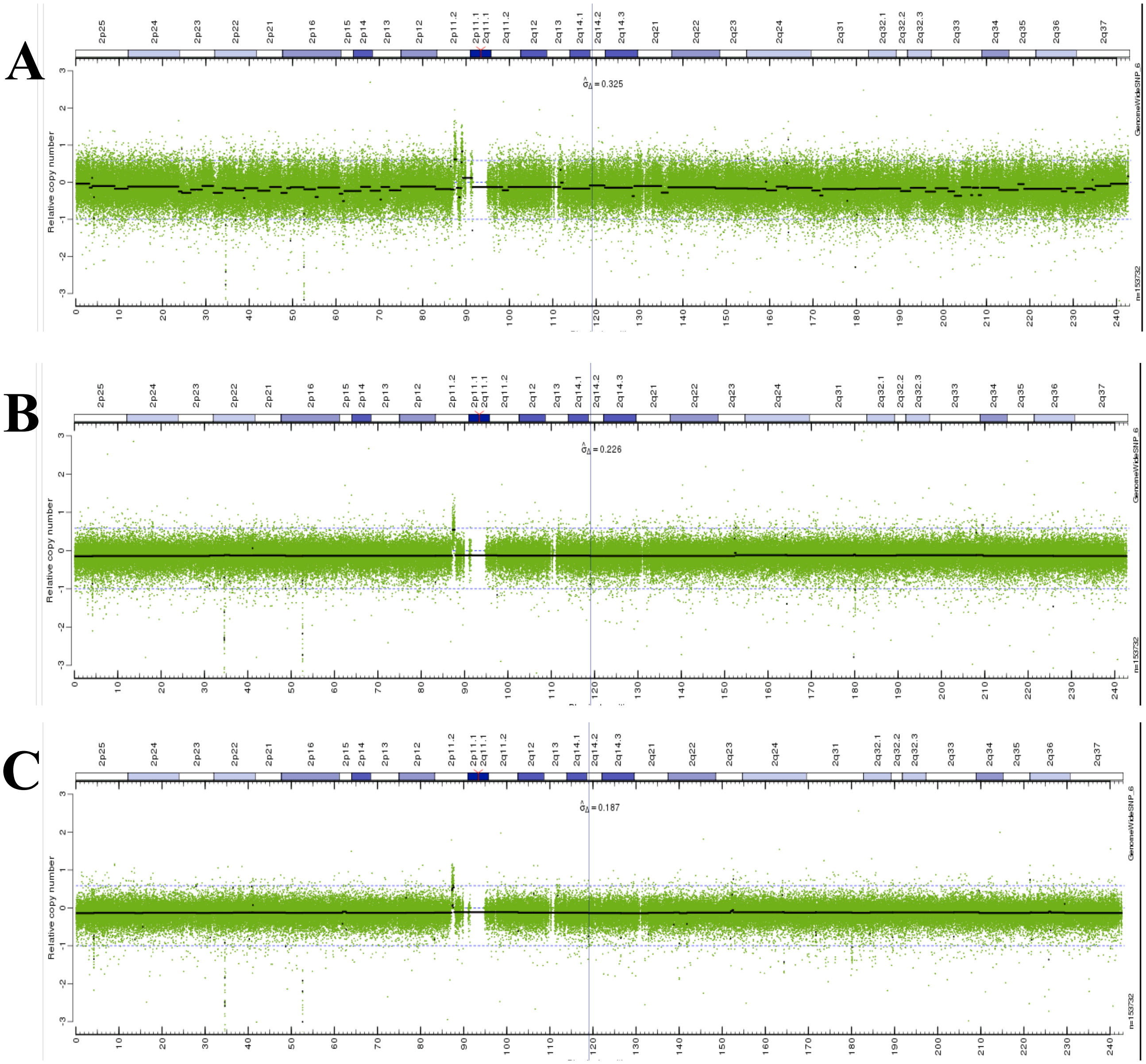
A
B
C
Relative copy number
GenomeWideSNP_6
n=153732
$\hat{\sigma}_\Delta = 0.325$
$\hat{\sigma}_\Delta = 0.226$
$\hat{\sigma}_\Delta = 0.187$
2p25
2p24
2p23
2p22
2p21
2p16
2p15
2p14
2p13
2p12
2p11.2
2p11.1
2q11.1
2q11.2
2q12
2q13
2q14.1
2q14.2
2q14.3
2q21
2q22
2q23
2q24
2q31
2q32.1
2q32.2
2q32.3
2q33
2q34
2q35
2q36
2q37
